# Hue selectivity from recurrent circuitry in *Drosophila*

**DOI:** 10.1038/s41593-024-01640-4

**Published:** 2024-05-16

**Authors:** Matthias P. Christenson, Alvaro Sanz Diez, Sarah L. Heath, Maia Saavedra-Weisenhaus, Atsuko Adachi, Aljoscha Nern, L. F. Abbott, Rudy Behnia

**Affiliations:** 1 Zuckerman Institute, Columbia University, New York, NY USA; 2 Center for Theoretical Neuroscience, Columbia University, New York, NY USA; 3 Department of Neuroscience, Columbia University Medical Center, New York, NY USA; 4 Janelia Research Campus, Howard Hughes Medical Institute, Ashburn, VA USA; 5 Present Address: Kavli Institute for Brain Science, Columbia University Medical Center, New York, NY USA

**Keywords:** Sensory processing, Neural circuits

## Abstract

In the perception of color, wavelengths of light reflected off objects are transformed into the derived quantities of brightness, saturation and hue. Neurons responding selectively to hue have been reported in primate cortex, but it is unknown how their narrow tuning in color space is produced by upstream circuit mechanisms. We report the discovery of neurons in the *Drosophila* optic lobe with hue-selective properties, which enables circuit-level analysis of color processing. From our analysis of an electron microscopy volume of a whole *Drosophila* brain, we construct a connectomics-constrained circuit model that accounts for this hue selectivity. Our model predicts that recurrent connections in the circuit are critical for generating hue selectivity. Experiments using genetic manipulations to perturb recurrence in adult flies confirm this prediction. Our findings reveal a circuit basis for hue selectivity in color vision.

## Abstract (web summary)

Christenson et al. describe hue-selective neurons in the fruit fly optic lobe. Using a connectomics-constrained model combined with genetic manipulations of the circuit, they show that recurrent connections are critical for hue selectivity.

## Main

Perceived features of sensory stimuli can differ from the physical attributes upon which they are based. These differences play a crucial role in how animals interact with the world around them. Understanding the neural circuit basis of the transformation from physical detection to perception is central to neuroscience. In color vision, the transformation from the spectral composition of light to derived color percepts is particularly dramatic. Reflectance spectra of objects, expressed as light intensities across a continuous range of wavelengths, are high dimensional, but this information is transmitted to the brain through a small number of photoreceptor channels; three for humans and four for flies. This drastic reduction generates, through processing downstream of the photoreceptors, the perceptual characterization of colors in terms of hue, saturation and brightness: the hue or tint of a color is related to the mean wavelength composition of the spectrum; saturation or degree of purity of a color is related to its variance; and brightness of a color is related to its total intensity. Using flies as a model system, our aim is to characterize the neural circuitry responsible for the processing that extracts these variables and underlies the transformation from spectral composition to perceptual colors.

Each ommatidium in the eye of the fruit fly *Drosophila melanogaster* has eight photoreceptors, labeled R1–R8. Color vision begins in yellow and pale variants of R7 and R8 photoreceptors (pR7, yR7, pR8, yR8) that express one of four opsins (Rh3, Rh4, Rh5 and Rh6, respectively) with peak sensitivities in the short ultraviolet (UV), UV, blue or green. As in trichromatic primates, photoreceptor activations are combined into color-opponent signals. In flies, these emerge at the terminals of the R7 and R8 photoreceptors, through both axo-axonal and interneuron-mediated interactions^1,2^. The fact that photoreceptor signals are compared in the form of color-opponent signals hinted at the possibility that flies may compute a high-dimensional (with up to four-dimensional, 4D) color space. We now ask how these opponent signals are further transformed downstream of the photoreceptors. Although the main postsynaptic partners of R7 and R8 have been identified^3,4^, and some have been implicated in color-guided behaviors^5,6^, the chromatic response properties of downstream neurons have not been described previously. Here we take advantage of the genetic tractability that fruit flies afford to measure visual responses of candidate neurons across fly color space. We show that the responses of three transmedullary (Tm) projection neurons downstream of R7s and R8s, Tm5a, Tm5b and Tm20, differ from and show nonlinear processing of their photoreceptor inputs, displaying hue-selective properties, with enhanced sensitivity to hue (including nonspectral hues) and/or decreased sensitivity to saturation.

Hue-selective neurons have been identified in primate visual cortex^7–12^. These neurons have responses that are narrowly tuned to specific spectral hues such as cyan, teal and orange, or nonspectral hues such as purple and magenta. Other neurons show selectivity for particular saturation or brightness levels^7,13^. Although primates are well suited for exploring the role of these neurons in perception, unraveling the circuit mechanisms that construct these signals is exceptionally challenging in such complex brains. Fortunately, the opportunity to study this question in circuits that are genetically accessible and mapped at the EM connectome level now exists in the fly brain. Given the similarities in neural processing discovered recently at the periphery in the form of color opponency^1,2^, we hypothesize that similarly convergent mechanisms will govern further transformation of chromatic neural signals in flies and nonhuman primates^14,15^.

To explore the circuit mechanisms behind hue-selective Tm responses, we analyzed the FAFB fly connectome data, using the neuron reconstruction environment FlyWire^16–18^, and constructed a circuit-level model based on this analysis and on our measurements of additional interneuron responses. This circuit model accurately matches the Tm responses we measured, and is consistent with the effects of experimentally silencing their synaptic outputs in the circuit. We find that recurrent connections within the circuit are critical for nonlinear hue-selective properties of Tm neurons. This work identifies biological mechanisms that govern the transition from sensory detection to perceptual representation.

## Results

### Hue selectivity is defined by the geometry of responses in color space

**Fig. 1:**
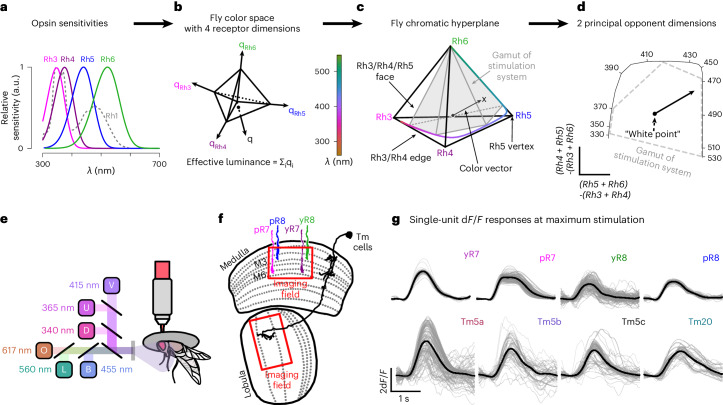
The *Drosophila* color space and experimental methods. **a**, Relative spectral sensitivity of opsins expressed in the fruit fly retina; data from ref. ^48^ fit with the equation from ref. ^49^. **b**, Fly color space defined by the photon captures *q* of Rh3, Rh4, Rh5 and Rh6 opsins. The luminance of a color is defined as the sum of opsin captures (∑_*i*_*q*_*i*_). **c**, Fly chromatic hyperplane defined by restricting the luminance to a constant value. In the chromatic hyperplane, there are four vertices, one for each opsin, six edges between pairs of opsins and four faces connecting three opsins. The gray box indicates the gamut of fly colors accurately reproducible with our stimulation system. Within the chromatic plane, the saturation of a color is defined as the distance of the stimulus from the fly’s effective white point (that is, the center of the tetrahedron). The hue of a color is defined by the angular direction of the color vector stretching from the white central point to a particular stimulus point. **d**, 2D projection of the fly color space onto the two color-opponent components: (Rh5 + Rh6) − (Rh3 + Rh4) and (Rh4 + Rh5) − (Rh3 + Rh6). **e**, Two-photon imaging setup. The fly is secured facing the LED setup, and LED sources are combined using a custom color mixer to form a single collimated full-field beam. D, deep UV; U, UV; V, violet; B, blue; L, lime; O, orange. **f**, Schematic of the fruit fly color circuit indicating the imaging fields used to record photoreceptors and interneurons in the medulla and Tm neurons in the lobula. **g**, Example d*F/F* traces of single regions of interest (ROIs; gray traces) in response to the neuron’s preferred stimulus. The black line indicates the mean response. a.u., arbitrary units. Source data

There are a number of ways to construct color spaces, some of which involve perceptual measurements^19^. To characterize the response properties of fly chromatic neurons, we define a fly color space on the basis of opsin captures. The fly eye has five opsins (Fig. 1a). Rh1, expressed in R1–6 photoreceptors, is broadband and mediates achromatic vision^20^. We focus on the other four opsins, Rh3–6, expressed in pale and yellow R7 and R8 photoreceptors (Fig. 1a). We computed photon captures for each opsin (relative to the background) to define a 4D fly color space (Fig. 1b). Any color stimulus can be mapped to a point in this space with coordinates defined by the photon captures. To isolate a subspace in which luminance is fixed, we constrain points to have equal summed photon captures across the opsins. This defines a three-dimensional (3D) subspace that has the shape of a tetrahedron (Fig. 1c). The central point in the tetrahedron, which corresponds to equal photon captures across all the opsins, defines the color white. Other colors are represented by vectors that project from this white point to the point defined by their opsin photon captures. Any non-white colors—including nonspectral colors that cannot be produced by any single wavelength of light—can be represented by such a color vector (Fig. 1c). We define hue and saturation at fixed luminance as a parameterization of points within this tetrahedron. Specifically, we define saturation *s* as the length of the color vector. Hue is defined by the two angles that specify the direction of the color vector in the 3D space of the tetrahedron (for example, the polar and azimuthal angles of a spherical coordinate system). Thus, fly vision has two hue angles rather than the single hue angle in trichromatic vision. As stated at the outset, hue, saturation and brightness, especially as applied to human vision, are often defined through perceptual measurements. It is important to note that, throughout, we define hue and saturation solely in terms of fly opsin photon captures. Similarly, we do not use the term brightness, but instead refer to the sum of the photon captures of all four opsins as luminance.

The preferred color tuning of a neuron in the fly color space is defined as the hue direction that produces the maximum response for a given saturation and luminance. We will model the response amplitude for a given neuron as dependent on the angle *θ* between the color vector describing a particular stimulus and the preferred color vector of that neuron (we used one angle instead of two as a simplification). The response amplitude also depends on the saturation *s*. The dependence of the responses of a neuron on these variables, which specifies the response pattern in color space, defines its chromatic properties. The 3D nature of the fly isoluminant color space makes it difficult to visualize color tuning. We therefore constructed a reduced two-dimensional (2D) representation of the color tetrahedron. We replotted the data projected onto the two directions defined by the two color-opponent directions: (Rh5 + Rh6) − (Rh3 + Rh4) and (Rh4 + Rh5) − (Rh3 + Rh6) captures^2^ (Fig. 1d).

Defining neural response geometries requires measurement of responses over a large portion of color space, which is challenging. In primates, approaches such as closed loop iso-response methods^8,21^ have been developed to tackle this problem. In *Drosophila*, we instead take advantage of genetically identified and accessible neurons to measure the responses of particular chromatic neuron types over nearly the entire fly color space by sampling across multiple single neurons and animals. Using the fly’s spectral sensitivities, we constructed a ‘gamut stimulus set’ that combines six full-field light-emitting diodes (LEDs; Fig. 1e) at different relative intensities to cover a range of calculated photon captures that span the available chromatic dimensions in flies. We restricted our analysis to stimuli that were close to a single isoluminant hyperplane (Fig. 1c). To achieve this, for each recording, we randomly sampled capture values within the gamut of the stimulation system (six LEDs) at a total relative capture of 5× the background capture, and fit the LED intensities using methods described in ref. ^22^ (Methods). In addition, to evaluate response properties across different luminance levels, we created a ‘contrast stimulus set’ consisting of increasing intensities of single and mixtures of LED flashes. We tested the response of each neuron to flashes of each single LED at contrast steps ranging from 0.1× to 3× the total background intensity. Similarly, we mixed LEDs to produce larger contrast steps (Methods). We used both stimulus sets to probe the response properties of neurons in the *Drosophila* chromatic circuit, including R7 and R8 axonal terminals in the medulla, the lobula terminals of the transmedullary neurons Tm5a, Tm5b, Tm5c and Tm20, which are the main projection neuron outputs of these photoreceptors^3^, as well as various medulla interneurons, using cell-type-specific two-photon imaging of GCaMP6f (Fig. 1e–g).

### The main downstream targets of R7 and R8 photoreceptors have diverse chromatic tuning properties that differ from their direct photoreceptor input

Light activation of opsins in the rhabdomeres of R7 and R8 photoreceptors is the first stage of chromatic encoding in the fly optic lobe. These, as represented by photon captures, correspond to the axes of the fly color space described in the previous section. The second stage involves the generation of color-opponent responses within the axonal terminals of these photoreceptors, through direct (axo-axonal) and indirect (through the horizontal cell Dm9) interactions^1,2^. As a result, although each photoreceptor only expresses a single opsin, their axonal responses reflect activation from multiple opsins. These axonal signals are then transformed further in the medulla where the main downstream targets of R7 and R8 photoreceptor axons have been identified^3^: yR7 axons target Tm5a, pR7 axons target Tm5b. Tm5c is downstream of either of the R8s, favoring yR8s over pR8s. Tm20 is downstream of either pR8 or yR8 axons in their home column (that is, the neural processing unit in the medulla from which they receive the highest number of synapses). The fact that different Tm20 neurons receive input exclusively from either pR8 or yR8 suggests that there may be two functional subtypes of Tm20s that are not distinguished by current genetic lines, a point we will return to in interpreting the results of our Tm20 measurements. We imaged the responses of the terminals of all four Tm neurons in the lobula to the gamut stimuli and compared them to the color-opponent axonal responses of their photoreceptor input.

**Fig. 2:**
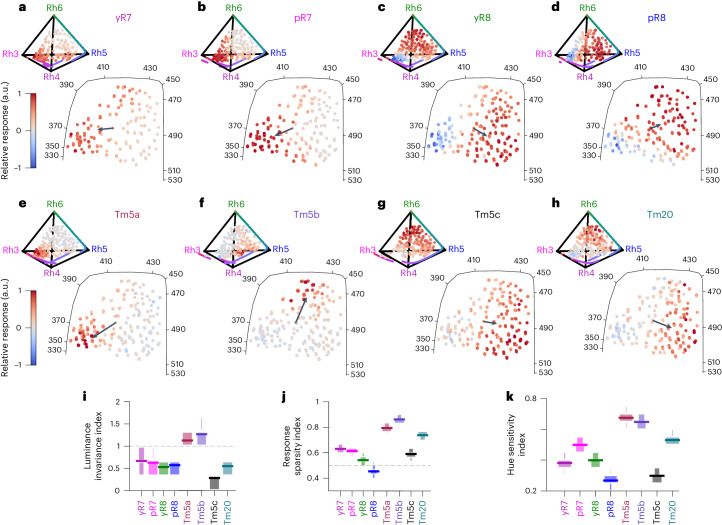
Responses of Tm neurons and their presynaptic photoreceptor axons in color space. **a**–**d**, Relative response amplitudes of yR7 (*n* = 195 ROIs, 8 flies), pR7 (*n* = 249 ROIs, 7 flies), yR8 (*n* = 396 ROIs, 8 flies) and pR8 photoreceptor (*n* = 182 ROIs, 4 flies) axons across the gamut of tested fly colors. Top, chromatic stimuli are represented as points in the chromatic tetrahedron, with the color of each point indicating the relative response of the indicated neuron to that stimulus. The colored line that spans the edges of the tetrahedron from Rh3 to Rh4, Rh5 and Rh6 corresponds to single wavelengths ranging from 300 nm to 560 nm (that is, the visible spectrum of the fly). Bottom, stimuli are represented as points in the 2D color-opponent space, with the color of each point indicating the relative response of the indicated neuron to that stimulus. The arrows correspond to the hue sensitivity vector (Methods). The solid black line corresponds to the single wavelength line, ranging across all single wavelengths from 330 nm to 530 nm. **e**–**h**, Same as **a**–**d** for Tm5a (*n* = 257 ROIs, 12 flies), Tm5b (*n* = 354 ROIs, 13 flies), Tm5c (*n* = 528 ROIs, 8 flies) and Tm20 (*n* = 289 ROIs, 16 flies). **i**, Mean luminance invariance indices for photoreceptors and Tm neurons (horizontal lines). The luminance invariance index is the ratio of the goodness of fit (*R*^2^) of a linear regression using only the chromatic dimensions and the *R*^2^ of a linear regression using only the achromatic dimension. The boxes correspond to a vertical histogram of the bootstrapped distribution. **j**, Mean sparsity indices for photoreceptors and Tm neurons (horizontal lines). A value of 0.5 corresponds to a uniform response distribution. The boxes correspond to a vertical histogram of the bootstrapped distribution. **k**, Mean hue sensitivity indices for photoreceptors and Tm neurons. The index quantifies tuning sharpness in stimulus space, with values near 1 indicating the neuron responds selectively to one stimulus direction, and values near 0 indicating equivalent responses across all stimulus directions. The boxes correspond to a vertical histogram of the bootstrapped distribution. Source data

The response patterns of photoreceptor axons in the tetrahedron are consistent with previous measurements^2^ (Fig. 2a–d). The responses of pR7, yR7, pR8 and yR8 axons peak in the short UV, UV, blue and green, respectively. pR8 and yR8 show clear opponent responses with inhibitory responses in the UV and long wavelength and UV, respectively. For R7s, opponency is not evident from the gamut responses because the background composition of the stimulus and GCaMP6f nonlinearity make it difficult to see inhibition from baseline. However, specific combinations of LEDs in the contrast stimulus are consistent with previous recordings (Extended Data Fig. 2a,b)^2^.

The responses Tm5a, Tm5b, Tm5c and Tm20 neurons peak at locations in color space that are different from their primary inputs (Fig. 2e–h). Tm5a responses are confined to the Rh3 vertex, with some activation along the Rh3/Rh6 edge, corresponding to the lower-left quadrant of the 2D projection (Fig. 2e). Tm5b is activated only along a narrow section of the Rh4/Rh5 edge of the tetrahedron, corresponding in 2D to activation confined to the upper-middle area of the space (Fig. 2f). Tm5c responses are broad with a preference close to the Rh6 vertex (Fig. 2g). Tm20 has the strongest activation within the Rh4/Rh5/Rh6 face, corresponding to photon captures localized in the bottom-right quadrant of the 2D space. (Fig. 2h).

**Extended Data Fig. 1:**
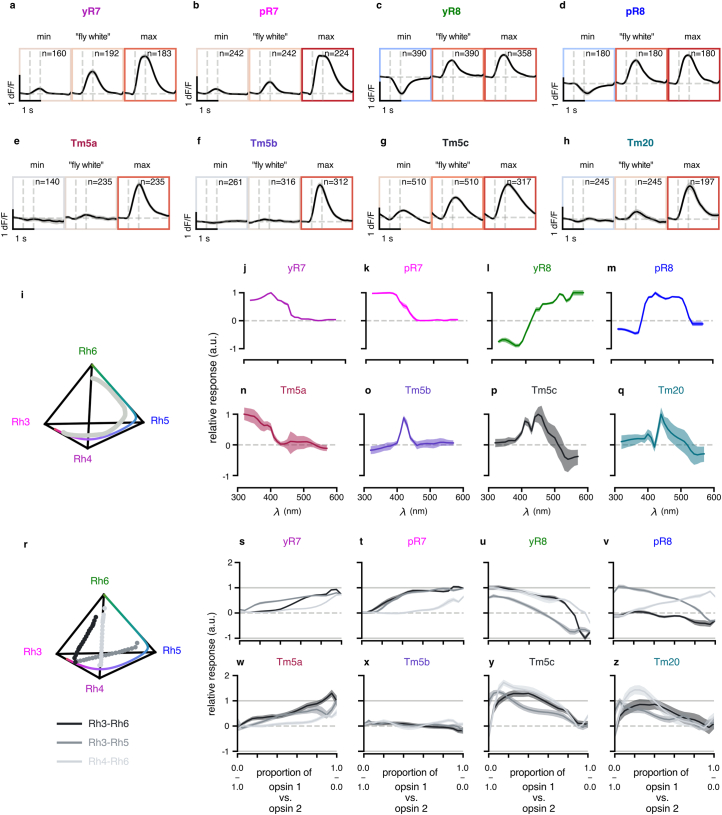
Additional response chromatic response profiles of photoreceptors and Tm neurons. **(a-h)** Min, ‘fly white’, and max responses of photoreceptors and Tm neurons in the chromatic hyperplane. Each of the three insets show dF/F responses across time from 0.5s before stimulus onset to 1.5s after stimulus offset, with onset and offset indicated by the dashed gray lines. The dashed gray horizontal line indicates the baseline dF/F. The insets show the average responses across recorded neurons around the location of the minimum amplitude, around the middle of the tetrahedron (‘fly white’), and around the location of the maximum response. The spherical volume used for averaging responses around the desired location for each plot has a radius of 0.05, with each edge of the tetrahedron having unit length. See Fig. 2a–h for the corresponding tetrahedra. The black line is the mean response and the gray shaded area indicates the 95% confidence interval of the bootstrapped mean response. **(i)** Approximate single wavelength line used for the interpolation of responses (line of gray scatter points). The colored line is the actual single wavelength line. **(j-q)** Interpolated single wavelengths for photoreceptors and Tm neurons. The colored line is the interpolated mean response. The shaded areas is the bootstrapped 95% percentile of the mean response. **(r)** Non-spectral lines used for interpolation of responses (lines of gray scatter points). The colored line corresponds to the single wavelength line. **(s-z)** Interpolated non-spectral responses of photoreceptors and Tm neurons. The colored lines correspond to the interpolated mean responses for different non-spectral lines (as indicated in the legend). The shaded areas correspond to the bootstrapped 95% percentile of the mean response. These are normalized to the max of the spectral line responses. Source data

**Extended Data Fig. 2:**
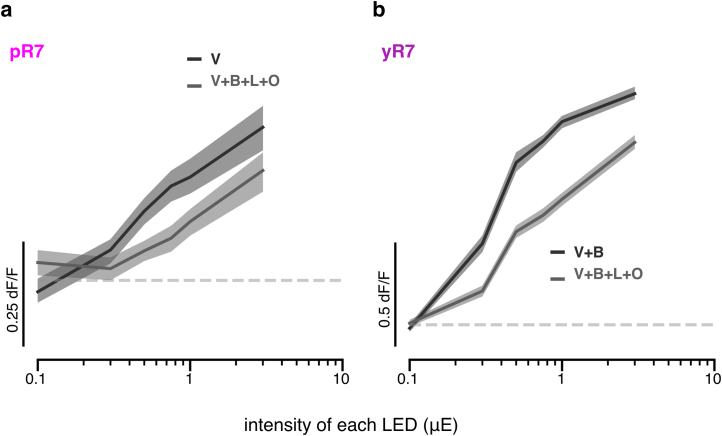
Responses of R7 photoreceptors to additive mixtures of particular LEDs. Presenting violet or violet+blue LEDs at increasing intensities on top of the adapted background produces stronger responses in pR7 (a) and yR7 (b), respectively, than presenting stimuli that add additional LEDs (that is with a higher overall intensity). The shaded area indicates the 95% confidence interval of the mean of the bootstrapped iterations of the data. Source data

To examine responses along one-dimensional paths through the 3D color space, we used radial basis functions to smoothly interpolate the responses from both the gamut and contrast stimulus sets (Methods). Along the single wavelength line, we obtained a spectral tuning curve (Extended Data Fig. 1i). For photoreceptors (Extended Data Fig. 1j–m), the results were, overall, consistent with our previous measurements^2^. In the case of Tm neurons, the spectral tuning curves peaked in the UV part of the spectrum for Tm5a (Extended Data Fig. 1n), violet for Tm5b (Extended Data Fig. 1o) and blue for Tm20 (Extended Data Fig. 1q). Tm5c was most sensitive to violet/blue/green light and had broader tuning than the other Tm neurons (Extended Data Fig. 1p). Tm5b, Tm5a and Tm20 neurons had narrower wavelength tuning than their presynaptic photoreceptor axon terminals (Extended Data Fig. 1j–q).

We also examined tuning to nonspectral colors^23–26^. An example in humans is purple, which is the result of multi-wavelength excitation of both S and L cones. Analogous colors for fruit flies correspond to exciting combinations Rh3–Rh6, Rh4–Rh6 or Rh3–Rh5 opsins (Extended Data Fig. 1r). We defined a neuron as non-spectrally tuned if its peak response is greater along a nonspectral line than along the single wavelength line. We observed nonspectral tuning in Tm5a, Tm5c and Tm20. Tm5a had its strongest response to a relative combination of 0.9 Rh3 and 0.1 Rh6 (Extended Data Fig. 1w). Tm5c had strong tuning along all the nonspectral lines we investigated, with peaks at 0.1 Rh3 and 0.9 Rh5, at 0.2 Rh4 and 0.8 Rh6, and at 0.3 Rh3 and 0.7 Rh6 (Extended Data Fig. 1y). Tm20 has its strongest response to a relative combination of 0.2 Rh4 and 0.8 Rh6 (Extended Data Fig. 1z). On the other hand, photoreceptor axon terminals and Tm5b have maximal responses to spectral colors (Extended Data Fig. 1s–v and Extended Data Fig. 1x).

If Tm5a, Tm5b and Tm20 responses were driven solely by their direct photoreceptor inputs, they would appear as a sign inverted version of the photoreceptor color-opponent responses because photoreceptors use the inhibitory transmitter histamine. The fact that this is not the case indicates that additional connections in the chromatic circuit are required to account for the Tm responses.

### Tm5a, Tm5b and Tm20 carry sparse chromatic signals and have narrow tuning

In contrast to the photoreceptor terminals and Tm5c, Tm5a, Tm5b and Tm20 do not show a strong response to stimuli close to the fly white point (Extended Data Fig. 1a–h). This suggested that they might have a reduced sensitivity to luminance. We computed a luminance invariance index, which is a measure of how well the responses can be explained by the luminance of the stimulus compared to the location of the stimulus in the color tetrahedron (Methods). Larger values correspond to more invariance (Fig. 2i). Photoreceptor terminals have luminance invariance indices around 0.5. Tm neuron indices vary across types, with Tm20 showing a luminance invariance index similar to that of the photoreceptor terminals, and Tm5c having a decreased luminance invariance. Tm5a and Tm5b, on the other hand, are less sensitive to luminance than the photoreceptor terminals with luminance invariance indices around 1.2.

A visual inspection of Fig. 2e,f,h suggests that Tm5a, Tm5b and, to a lesser degree, Tm20 respond to fewer stimuli than their photoreceptor inputs. To quantify this, we calculated a response sparsity index, which is a measure of responsiveness to a stimulus set defined as the area under a normalized cumulative histogram of responses (Methods). A uniform distribution of responses would yield a sparsity index of 0.5 for this measure. Larger values correspond to sparser response distributions (Fig. 2j). Tm5b has the sparsest response profile, with a sparsity index of 0.85, followed closely by Tm5a and Tm20, with indices of 0.8 and 0.75, respectively. Tm5c, in contrast, has a profile that is similar to those of photoreceptor axonal responses with an index around 0.6.

Increased sparsity of Tm5a, Tm5b and Tm20 responses could be the result of a narrower tuning of these neurons compared to photoreceptor axons. To quantify this, we calculated a hue sensitivity index, which measures the concentration of responses in color space around the preferred tuning vector (Methods). An index of 1 corresponds to a neuron that only responds to one specific stimulus direction. A value of 0 corresponds to a neuron that responds to all stimulus directions equally (Fig. 2k). We find that Tm5a and Tm5b have a hue sensitivity index of 0.65, higher than the axonal terminals of R8 and R7 photoreceptors, which are in between 0.25 and 0.5. Tm20’s index is 0.55, intermediate between Tm5a/Tm5b and photoreceptor axons, related to the more moderate sparsity of its response pattern. Tm5c’s index is in the same range as the photoreceptor axonal terminals, consistent with the broad tuning of this neuron.

Our results to this point show that, downstream of photoreceptors, visual information is reformatted into four parallel channels that convey distinct types of information. Tm5a and Tm5b have narrowly tuned responses to specific spectral and nonspectral colors, with reduced sensitivity to luminance. Tm20 has a sparse response pattern, but is less narrowly tuned to hue than Tm5a and Tm5b, and has a sensitivity to luminance similar to the photoreceptor terminals. In contrast, Tm5c is broadly tuned in color space and has a heightened sensitivity to luminance. The connectomic analysis discussed below indicates that, despite its projection to the lobula, Tm5c is equivalent to an interneuron, acting mostly locally within this circuit. This is consistent with the fact that, unlike Tm5a, Tm5b and Tm20, which are predicted to be cholinergic, Tm5c is glutamatergic^27^. For these reasons, we focus on the response properties of Tm5a, Tm5b and Tm20 in the remainder of our analysis and discuss the role of Tm5c as a source of recurrent connections later in the paper.

### Tm5a, Tm5b and Tm20 responses are nonlinear

The responses of Tm5a, Tm5b and, to a lesser extent, Tm20 have properties that are reminiscent of nonlinear hue-selective neurons that have been identified in the cortex of trichromatic primates^7–12^. In the remaining sections, we focus on establishing this quantitatively, and then we explore the circuit mechanisms underlying the signal transformation that leads to hue selectivity in these Tm neurons.

**Fig. 3:**
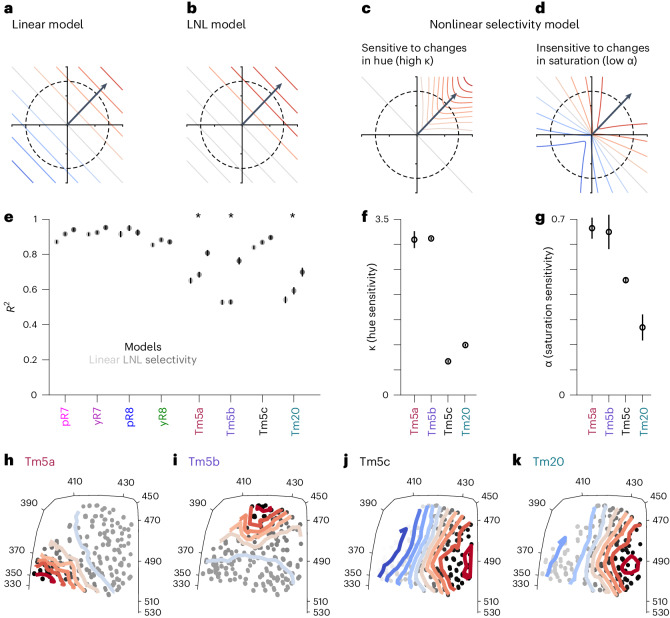
Tm5b, Tm5a and Tm20 responses are nonlinear. **a**–**d**, Iso-response contours (red and blue denote activation and inhibition, respectively) for a hypothetical neuron that is linear (**a**), LNL (**b**), hue-sensitive (**c**) or saturation-insensitive (**d**). In the LNL model, a tanh function is fit to account for response saturation. In contrast, our hue-selectivity model fits two parameters, *κ* and *α*, that account for a nonlinear sensitivity to hue and saturation, respectively. **e**, Comparison of mean *R*^2^ values for the linear (light gray), LNL (gray) and the nonlinear selectivity (dark gray) model fits to the data for photoreceptors and Tm neurons. Mean *R*^2^ values were obtained using all the data. The error bars indicate the 95% confidence interval of fitting the models to bootstrap iterations of the data. The asterisk indicates a significant difference in the *R*^2^ between the nonlinear selectivity model and the linear or LNL model as determined by their confidence intervals. **f**, *κ* values from fitting the nonlinear selectivity model. Mean *κ* values were obtained using all the data. The error bars indicate the 95% confidence interval of *κ* after fitting the nonlinear selectivity model to the bootstrapped distribution of mean responses. **g**, *α* values from fitting the nonlinear selectivity model. Mean *α* values were obtained using all the data. The error bars indicate the 95% confidence interval of *α* after fitting the nonlinear selectivity model to the bootstrapped distribution of mean responses. **h**–**k**, The iso-response contours (colored lines) of Tm neurons as predicted by the nonlinear selectivity model in the 2D color-opponent space. The amplitude of the responses predicted by the nonlinear selectivity model to individual stimuli for the Tm neurons are represented as single points on a gray scale in the plot. Source data

A fundamental property of hue-selective neurons is the nonlinearity of their responses with respect to their photoreceptor inputs. Different forms of selectivity can be extracted from the geometry of response patterns in color space. In a 2D color space, such as our color-opponent space, a linear response corresponds to a pattern of evenly spaced parallel iso-response contours that are orthogonal to a preferred tuning direction (Fig. 3a). Along a circle of constant saturation, this tuning has a cosine shape, peaking at the preferred direction of tuning. This defines what we call the linear model. Applying an output nonlinearity (such as tanh function) in the context of a linear–nonlinear (LNL) model can modify response amplitudes and contour spacing, but it cannot change the shape of the iso-response contours (Fig. 3b). Increased sensitivity to hue corresponds to bending iso-response contours into U-shaped curves, without changing the spacing between them (Fig. 3c). Reduced sensitivity to saturation produces iso-response lines that are rotated toward the preferred direction of tuning, also forming U-shaped curves (Fig. 3d). Both of these nonlinear mechanisms generate responses that are confined in color space and thus hue selectivity. Within this framework, we therefore ask two questions: (1) are the responses of Tm neurons linear functions of their photoreceptor inputs and, if not, (2) can we express their responses as a function of photoreceptor photon captures and quantify their increased sensitivity to hue and decreased sensitivity to saturation.

**Extended Data Fig. 3:**
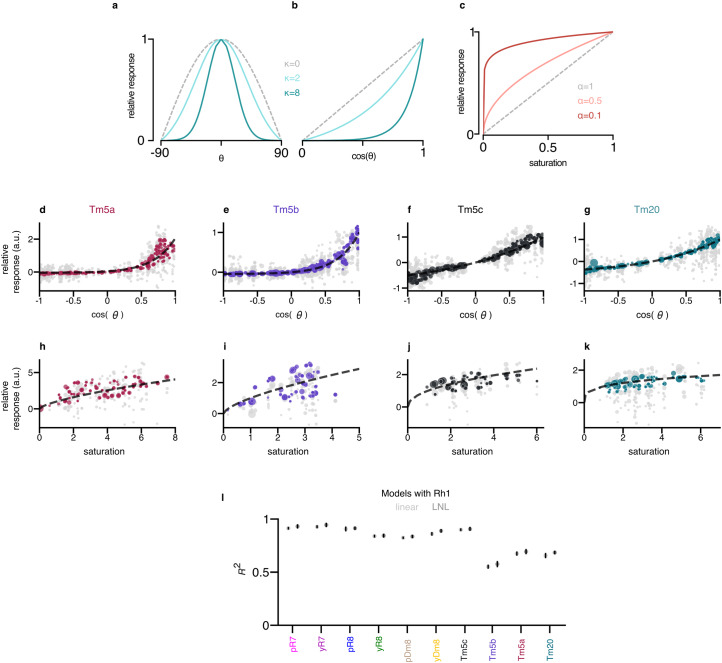
Additional analysis of the fitted nonlinear selectivity models. **(a-b)** Different sensitivities to the hue angle relative to the preferred direction of tuning for fixed saturation in the nonlinear selectivity model. The gray dashed line shows the linear case and the colored lines show increasing sensitivity to hue, as indicated by an increase in *κ*. **(c)** Different sensitivities to saturation for a fixed hue angle. The gray dashed line shows the linear case and the color lines show decreasing sensitivity to saturation, as indicated by a decrease of the *α* parameter in the nonlinear selectivity model. **(d-g)** Relative responses of the non-linear selectivity model (colored) and the data (gray) across $\cos (\theta )$, the cosine of the angle between the preferred tuning direction and the stimulus direction. The dashed line is the fitted curve using the obtained *κ*. **(h-k)** Relative responses of the non-linear selectivity model (colored) and the data (gray) across saturation values. The dashed line is the fitted curve using the obtained *α*. Only responses with a $\cos (\theta )$ greater than 0.5 are shown. **(l)** Mean *R*^2^ values using a linear model with the additional Rh1 feature (that is the calculated excitation of R1-6 photoreceptors) for all neurons. The error bars indicate the 95% confidence interval of fitting the models to the bootstrapped iterations of the data. Source data

In contrast to their photoreceptor inputs^2^, Tm5a, Tm5b and Tm20 are not well fit by a linear model (Fig. 3e). Including Rh1, which is known to provide some input to color pathways^28,29^, does not improve the linear fits, except for a slight increase in the *R*^2^ for Tm20 (Extended Data Fig. 3l). Including an output nonlinearity in the form of a tanh function only improves fits marginally in some cases (Fig. 3e).

These results imply that some form of nonlinear processing of photoreceptor axon excitation gives rise to the responses of Tm5a, Tm5b and Tm20 neurons. We therefore constructed a nonlinear model that, as a function of its parameters, allowed for both sharpening of sensitivity to hue angle and decreased sensitivity to saturation. In this nonlinear selectivity model, the response depends on the cosine of the angle between the stimulus color vector and the neuron’s preferred color direction, *θ*, and on the saturation level, *s*. To introduce nonlinear dependencies, the model response depends on a von Mises function of the linear hue selectivity, $\cos (\theta )$, with a variable *κ* characterizing the narrowness of the hue selectivity. It also depends on the saturation parameter raised to the power *α*. The values *κ* → 0 and *α* = 1 result in a linear response, while increasing *κ* tightens the model’s color tuning, and decreasing *α* reduces its dependence on saturation. The curves shown in Fig. 3c,d were generated by this model, which we call the nonlinear selectivity model.

The nonlinear selectivity model did not improve fits for photoreceptor axons or Tm5c beyond those of the linear model, but it generated much better fits for Tm5a, Tm5b and Tm20 (Fig. 3e). The fitted *κ* for Tm5a and Tm5b stand out at around 3, indicating narrow hue tuning, values much higher than those of Tm5c and Tm20, which are between 0 and 1 (Fig. 3f). In contrast, the estimated *α* value for Tm20 is low, around 0.25, while those of Tm5a and Tm5b are higher, around 0.7, and that of Tm5c is intermediate, around 0.45 (Fig. 3g), indicating various degrees of reduced sensitivity to saturation. To get a better intuition for the output of the nonlinear selectivity model, we plotted iso-response contours obtained with the nonlinear selectivity model in the 2D color-opponent space. In the case of Tm5c, the responses (mostly) varied uniformly along their preferred tuning axis and the iso-responses contours were parallel to each other (particularly around the white point), reflecting its linear properties (Fig. 3j). In contrast, Tm5a and Tm5b showed curved iso-responses contours around their preferred tuning (Fig. 3h,i). In the case of Tm20, iso-response contours appeared curved where responses are positive, and more spaced out in the inhibitory region (Fig. 3k).

To visualize more directly the tighter color tuning of the Tm neurons, we projected their responses onto a 2D plot of response versus $\cos (\theta )$ (compare Extended Data Fig. 3b with Extended Data Fig. 3d–g). The scatter in these plots reflects both response variability and the fact that we have projected responses across a range of saturations onto this single graph. In a linear model, the responses in these plots would be linear in $\cos (\theta )$, as is the case for Tm5c. Instead, we saw responses, especially for Tm5a and Tm5b, that rose sharply near the preferred color direction ($\cos (\theta )=1$). Similarly, we can visualize the tuning of Tm neurons with respect to saturation for stimuli that are close to the preferred direction of tuning ($\cos (\theta ) > 0.5$) (compare Extended Data Fig. 3c with Extended Data Fig. 3h–k). The responses were almost flat for Tm20 (Fig. 3k).

Our results suggest that Tm5a, Tm5b and Tm20 responses are nonlinear, that Tm5a and Tm5b are hue selective, and that Tm20 is fairly invariant to saturation. We mentioned earlier that there may be two subclasses of Tm20 neurons, and the responses of Tm20 could be interpreted as consisting of two hue-selective clusters. Thus, it may be possible that Tm20, when properly split into subtypes, will, along with Tm5a and Tm5b, comprise four types of hue-selective neurons. We will investigate this hypothesis further using our circuit model.

### Connectomics reconstruction reveals a highly recurrent circuit between Tm output neurons

Our nonlinear selectivity model is useful for quantifying hue selectivity but is not informative when it comes to determining neural circuit mechanisms that underlie these signals. To identify the circuit motifs that support the nonlinear selectivities in Tm5a, Tm5b and Tm20, we combined connectomics-constrained modeling with genetic manipulations of the circuit.

**Extended Data Fig. 4:**
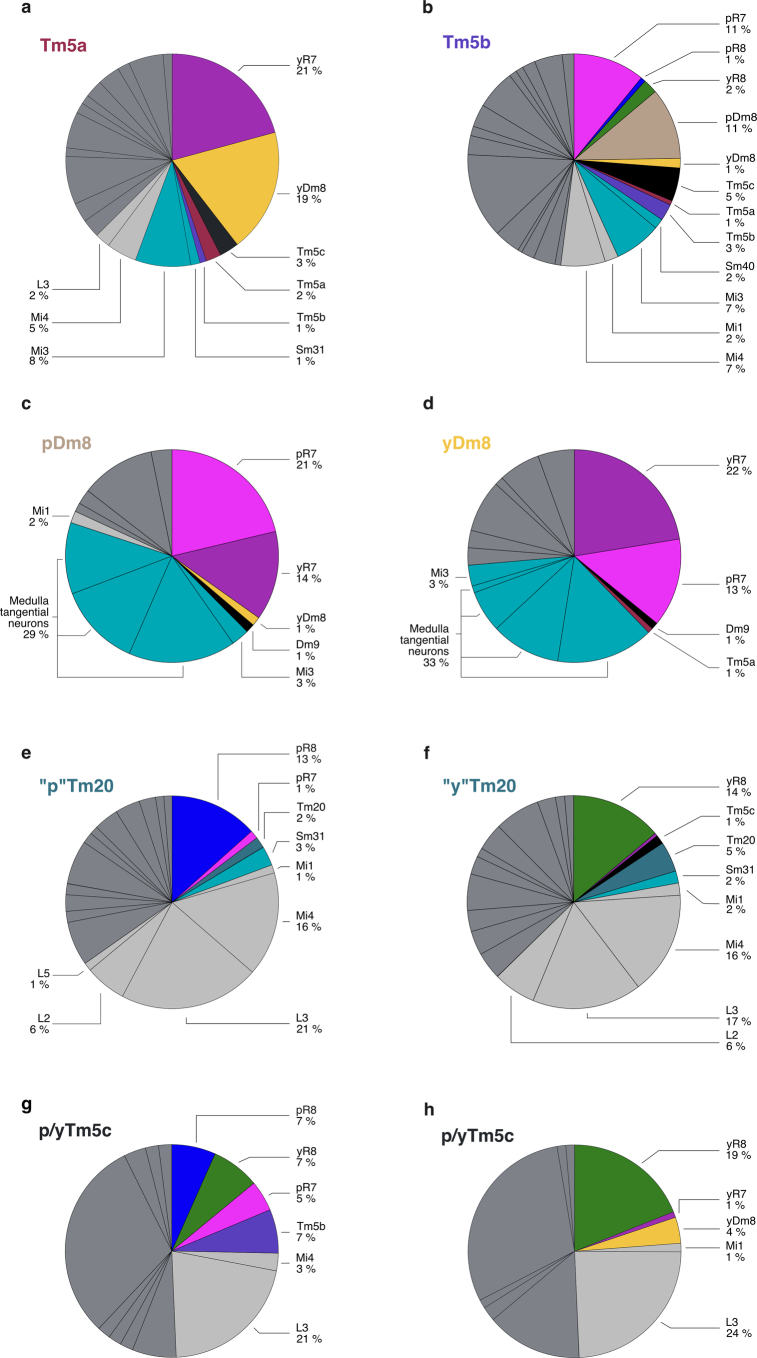
Presynaptic connectivity of Tm5a, Tm5b, Tm5c, Tm20 and y/pDm8. **(a-h)** Fraction of total inputs ( > 2 synapses) to Tm5a, Tm5b, Tm5c, Tm20, pDm8 and yDm8 obtained from EM reconstruction and identification of presynaptic partners in the medulla and the lobula. **(a,b)** Averaged fraction of inputs of two reconstructed Tm5a and two Tm5b cells respectively. **(c-h)** Input fractions onto individual neurons. Colored wedges represent inputs from neurons addressed in this study (R7s, R8s, Dm8s, Tm5s and Tm20s). Teal specifically denotes recurrent inputs (Mi3 and medulla tangential neurons). Light gray wedges correspond to indirect input from R1-6 photoreceptors and dark gray are all other inputs. The complete presynaptic connectivity of these cells is detailed in supplementary tables (Supplementary Tables 3–12). Source data

Information about the identity of the presynaptic partners of the Tm neurons was partially determined in work by Takemura et al.^4^, where synaptic circuits of seven columns in the medulla were reconstructed from electron microscopy (EM). However, this dataset included many unidentified neurons, often those that span more than seven columns and did not include lobula inputs/outputs. We therefore performed independent tracing of synaptic inputs and outputs of the nonlinear Tm neurons, Tm5c, as well as the amacrine-like neurons yDm8 and pDm8, major inputs to Tm5a and Tm5b, respectively (Extended Data Fig. 4 and Supplementary Table 1)^16–18^. Overall, our results are consistent with the seven-medulla column dataset, but they offer new insights into the connectivity of this circuit.

The two major inputs to Tm5a are their home column yR7 and yDm8 (≈20% each; Extended Data Fig. 4a). An equivalent circuit exists upstream of Tm5b, with pR7 and pDm8 being the major inputs (≈12% each; Extended Data Fig. 4b). pDm8 and yDm8 are amacrine-like interneurons^6,30^. The two subtypes are defined by the identity of the strongest single R7 subtype input in their home column^31,32^. As expected from previous work, Dm8 neurons synapse onto each other (Extended Data Fig. 4c,d)^28^ and receive indirect R1–6 inputs^28,29^. The rest of the inputs are dominated by large medulla tangential neurons that connect neighboring Dm8s. Tm5a and Tm5b get indirect inputs from R1–6 (through Mi4 ≈ 6%). Both also get direct inputs from themselves, each other and Tm5c. Together, these inputs correspond to approximately 9–10% of total inputs to Tm5a and Tm5b.

Connectivity onto Tm20 is simpler (Extended Data Fig. 4e,f). The major inputs to Tm20 are either a single yR8 or single pR8 (≈13%), as well as lamina monopolar cells (≈17–30%) from the same ommatidial column. The latter should provide OFF contributions from R1–6, but Tm20 also receive ON contributions from Mi4 and Mi1 (≈17%). These neurons together make up more than half of all the inputs to Tm20. Tm20 neurons also get input from themselves (≈2–5%). We did not see any obvious differences in input connectivity between Tm20 neurons downstream of yR8 and pR8.

We also reconstructed Tm5c inputs (Extended Data Fig. 4g,h). The two neurons we reconstructed received either purely y column input (yR8 and to a lesser extent yR7 and yDm8) or a mixture of yR8 and pR8, and some R7. Together with the lamina monopolar cell L3, these constitute a little under half of all inputs to Tm5c. The Tm5c downstream of y columns receives inputs from yDm8.

**Extended Data Fig. 5:**
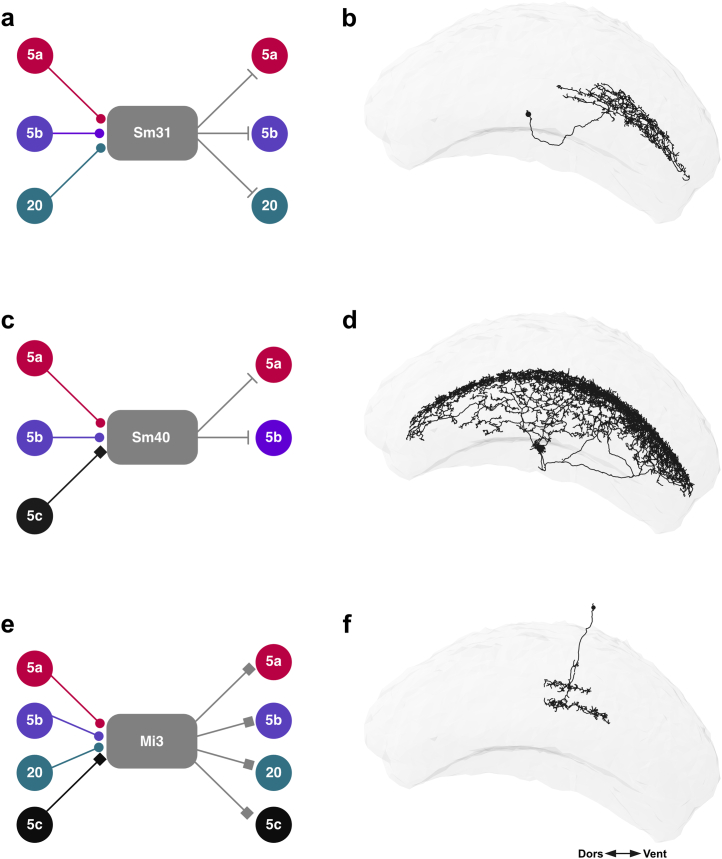
Putative chromatic recurrent circuits in the medulla between Tm cells and local interneurons. Our EM reconstructions revealed the presence of three types of interneurons that establish synaptic recurrence between different Tms. **(a,b)** The medulla tangential neuron Sm31^33^, ramifying across medulla layers M6 and M8, was found as both pre and postsynaptic to Tm5a and Tm20. Loose reconstruction of inputs and outputs from five of these cells confirmed Tm5a and Tm20 connectivity and showed that this cell type is also pre and post synaptic to Tm5b cells. Somas are located in the medial-anterior side of the medulla. Neurotransmitter prediction suggests they are GABAergic. **(c,d)** Tm5a, Tm5b and Tm5c send outputs to a putatively GABAergic large tangential neuron, Sm40^33^ (also named CB3841^18^), that expand its dendritic tree across medulla M6 layer and sends outputs to Tm5a and Tm5b. **(e,f)** Combining our Tm reconstructions and the seven column EM dataset^4^ we observed that Mi3 is both pre and postsynaptic to Tm5a, Tm5b, Tm5c and Tm20. **(b,d,f)** Single cell skeletons of Sm31, Sm40 and Mi3 (Flywire segments ids 720575940644160968, 720575940625550823, 720575940623904136 respectively). Source data

Because of the prevalence of self-connections in the circuit, we also reconstructed outputs of the Tm neurons (Supplementary Table 2). This analysis revealed that both Mi3 and several of the large medulla tangential neuron inputs (Sm31 and Sm40)^33^ found in the input connectivity to Tm neurons are also outputs of the Tm neurons (Extended Data Fig. 5). Tm neurons are therefore connected by a recurrent network that involves both of these cell types.

**Fig. 4:**
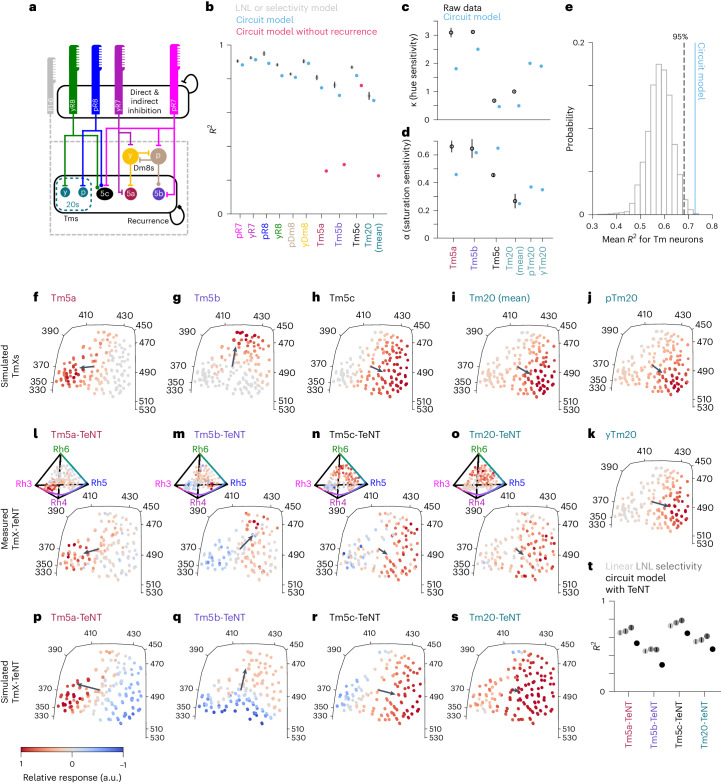
Recurrence is required for hue selectivity of Tm5a, Tm5b and Tm20. **a**, Schematic of the medulla color circuit. A connection with a flat bar is inhibitory, a connection with a filled circle is excitatory, and a connection with a filled square can be either sign. R7 and R8 axons are mutually inhibited (circular arrow with flat bar ending), giving rise to opponent responses. R1–6 provide indirect connections to Dm8s and Tm neurons. The connection to Dm8s is excitatory, while the sign to Tm neurons is not fixed but determined by the fitting procedure. There are monosynaptic (direct) and disynaptic (indirect) recurrent connections between Tm neurons (black circular arrow). **b**, Mean *R*^2^ for the LNL or nonlinear selectivity model as in Fig. 3e and Extended Data Fig. 3a, the circuit model when fitting to all wild-type data (cyan), and when removing recurrence in Tm neurons (red). **c**, Mean *κ* values from fitting the nonlinear selectivity model to the circuit model responses (colored) and the raw data (black). **d**, Mean *α* values from fitting the nonlinear selectivity model to the circuit model responses (colored) and the raw data (black). **e**, Distribution of mean *R*^2^ values for Tm neurons using random Tm input weights for the circuit model. The dashed line indicates the 95th percentile of the distribution, and the solid colored line indicates the mean *R*^2^ value using the synaptic counts as weights. **f**–**k**, Predicted responses of all Tm neurons according to the circuit model with recurrence. Tm20 (mean) was calculated using a weighted average of the simulated pTm20 and yTm20 responses according to the proportion of pale (one-third) and yellow columns (two-thirds). **l**–**o**, Measured Tm responses in flies where TeNT is expressed in each of the respective neurons to silence their outputs. Tm5a-TeNT (*n* = 138 ROIs, 11 flies), Tm5b-TeNT (*n* = 163 ROIs, 14 flies), Tm5c-TeNT (*n* = 296 ROIs, 5 flies) and Tm20-TeNT (*n* = 188 ROIs, 10 flies). **p**–**s**, The simulated responses of Tm-TeNT flies in the circuit model after refitting the offset and gain parameters. In **f**–**s**, the arrow corresponds to the hue sensitivity vector (Methods). **t**, Mean *R*^2^ values for the Tm-TeNT flies fitted to the linear (light gray), LNL (gray) and selectivity (dark gray) models, as well as the fit for the predicted response in the TeNT flies in the circuit model (black). The offset and gain parameter for each perturbed Tm neuron (that is, a total of two parameters) were the only free parameters when fitting the circuit model to the TmX-TeNT flies. In **b**–**d** and **t**, we show mean values and error bars indicate the 95% confidence interval of fitting the models to bootstrap iterations of the data. Source data

Overall, the picture that emerges from this connectomics reconstruction is that of a feedforward circuit driven largely by R7s and R8s, combined with a highly interconnected circuit, where both direct and indirect connections contribute to recurrence. This recurrent circuitry, which exists at multiple levels mostly in the form of lateral connections, is a factor that was underestimated in the original seven-column dataset^4^, because of its small volume. A simplified version of this chromatic circuit is depicted in Fig. 4a.

### yDm8 and pDm8 responses are linear but different from their primary inputs

**Extended Data Fig. 6:**
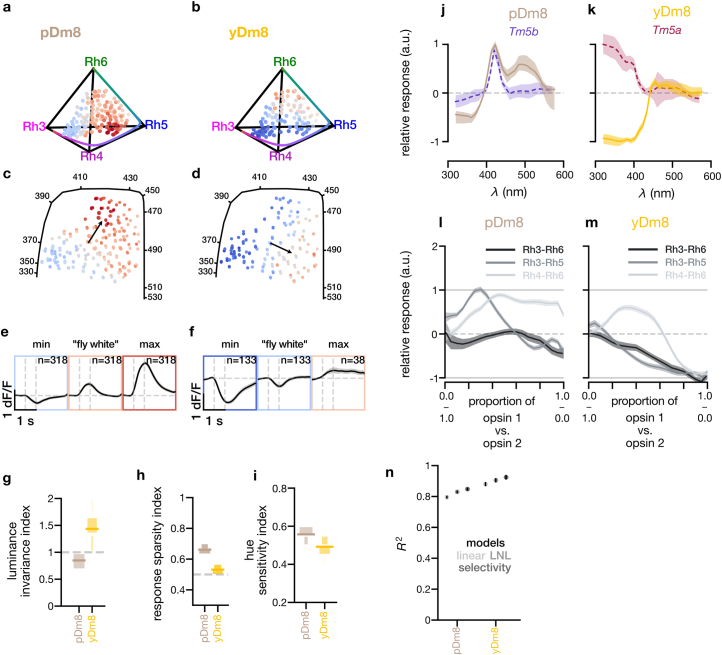
Chromatic response properties of pDm8 and yDm8 neurons. **(a-d)** Relative response amplitude of pDm8 (n= 333 ROIs, 7 flies) and yDm8 (n= 135 ROIs, 3 flies) across the gamut of tested fly colors. **(a,b)** Chromatic stimuli are represented as points in the chromatic tetrahedron, with the color of each point indicating the relative response of the indicated neuron to that stimulus. The color line that spans the edges of the tetrahedron from Rh3 to Rh4, Rh5 and Rh6 corresponed to the single wavelengths ranging from 300nm to 560nm. **(c,d)** Stimuli are represented as points in the 2D color opponent space, with the color of each point indicating the relative response of the indicated neuron to that stimulus. The solid black line corresponds to the single wavelength line, ranging across all single wavelengths from 330 to 530nm. **(e,f)** Min, ‘fly white’, and max responses of pDm8 and yDm8 as in Extended Data Fig. 1a-h. **(g)** Mean luminance invariance indices for Dm8 neurons (horizontal line). The boxes correspond to a vertical histogram of the bootstrapped distribution. **(h)** Mean sparsity indices for Dm8 neurons (horizontal line). The boxes correspond to a vertical histogram of the bootstrapped distribution. **(i)** Mean hue sensitivity indices for Dm8 neurons (horizontal line). The boxes correspond to a vertical histogram of the bootstrapped distribution. **(j,k)** Interpolated single wavelengths for Dm8s. The colored line corresponds to the interpolated mean response. The shaded areas correspond to the bootstrapped 95% percentile of the mean response. Tm5b and Tm5a single wavelength responses from Extended Data Fig. 1n,o are overlaid on pDm8 and yDm8 for comparison, respectively. **(l,m)** Interpolated non-spectral responses of Dm8s. The colored lines correspond to the interpolated mean responses for different non-spectral lines (as indicated in the legend). The shaded areas correspond to the bootstrapped 95% percentile of the mean response. **(n)** Comparison of different mean *R*^2^ values using standard linear regression, the LNL model, and the selectivity model for Dm8s. Mean *R*^2^ values are obtained using all the data and the error bar is the 95% confidence interval of fitting the models to bootstrap iterations of the data. Source data

Synaptic connectivity highlights pDm8 and yDm8 as important nodes in the color processing circuit. To incorporate them into our circuit-constrained model, we measured their responses to the same stimulus set used for the photoreceptors and Tm neurons. pDm8 was excited by combinations of Rh4, Rh5 and Rh6 activation, with larger responses in the area of the tetrahedron corresponding to violet (Extended Data Fig. 6a,c,e). It also had inhibitory responses along the Rh3/Rh4/Rh6 face of the tetrahedron. yDm8 was broadly inhibited, along the Rh3/Rh4, Rh3/Rh6 and Rh4/Rh5 edges. It had positive responses close to the Rh6 vertex and along the Rh5/Rh6 edge (Extended Data Fig. 6b,d,f). pDm8 and yDm8 responses peaked along the single wavelength line (Extended Data Fig. 6j,k), in agreement with previous recordings using isoquantal single wavelength stimuli^28^, and were spectral (Extended Data Fig. 6l,m). The overall direction of the tuning of Tm5b aligned with that of pDm8 (Extended Data Fig. 6k). However, Tm5a tuning was flipped relative to yDm8 tuning (Extended Data Fig. 6j), suggesting that pDm8 forms excitatory synapses with Tm5b and inhibitory synapses with Tm5a. This is plausible because Dm8 is glutamatergic^28,34^, which, in fruit flies, can be either excitatory or inhibitory depending on the postsynaptic receptor. The luminance sensitivity indices for pDm8 and yDm8 were 0.7 and 1.5 (Extended Data Fig. 6g), respectively. Their sparsity indices were 0.65 and 0.55, respectively (Extended Data Fig. 6h), and their effective hue sensitivity index was around 0.5 (Extended Data Fig. 6i). Both pDm8 and yDm8 responses were accurately predicted by a linear model (Extended Data Fig. 6n); adding an output nonlinearity did not improve the fits. The nonlinear selectivity model did not improve fits either.

### Recurrence is required for hue selectivity in Tm5a, Tm5b and Tm20

Which features of the circuit contribute to hue selectivity? To answer this question, we built a recurrent circuit model constrained by connectomics data that can account for the observed responses in all the neurons we imaged, that is, color-opponent photoreceptor terminals, interneurons and Tm neurons. This connectomics-constrained circuit model is based on known direct and indirect connections in the medulla connectome (Fig. 4a). In this model, each neuron, including Tm neurons, integrates its inputs linearly and applies a tanh output nonlinearity with an offset parameter that determines when the neuron saturates. This is in contrast to the nonlinear selectivity model described in Fig. 3, where Tm neurons explicitly integrate photoreceptor signals nonlinearly (input nonlinearity), and different from the LNL model, where inputs were not subject to recurrent interactions. We fit the connectomics-constrained circuit model to the responses of all neurons in three stages: first the photoreceptor–Dm9 circuit, then the Dm8 recurrent circuit, and finally the Tm neuron recurrent circuit. We included two subtypes of Tm20 neurons in the model to account for the two Tm20 input varieties (yR8 and pR8) and to test the hypothesis that our fly line superimposes the responses of two more highly hue-selective neuron subtypes.

In ref. ^2^, we showed that opponent responses in photoreceptor axons can be accurately modeled using EM-derived synaptic counts as quantitative estimates of the synaptic weights. Thus, we fixed the weights between R7s, R8s and Dm9s to values proportional to the connectome synapse counts and included two gain parameters—one common gain for all of the photoreceptor axons, and one for the Dm9 interneuron. A third gain parameter was added to vary the overall gain of R7 and R8 rhabdomeric inputs.

The second stage of the circuit corresponds to the indirect connection between R1–6 and Dm8s, direct connections from R7s onto Dm8s, and recurrent connections between Dm8s. We fit the proportion of R1–6 inputs and the recurrent connections between Dm8s, as well as a common gain parameter for pDm8 and yDm8. We constrained R1–6 inputs to be positive and R7 inputs and indirect connections to be negative, as shown previously^28,29^.

At the last stage of the circuit, corresponding to the feedforward and recurrent connections onto Tm neurons, we fixed the feedforward weight from R7s, R8s and Dm8s, as well as the recurrent weights between Tm neurons according to the relative synaptic counts of our EM reconstruction. We explicitly modeled yTm20 and pTm20, as defined by the type of R8 input that they receive according to the connectome. The total weight for each recurrent connection included both direct (monosynaptic) and indirect (disynaptic) connections as identified in the EM reconstruction. We fit the proportion of the R1–6 inputs onto each Tm neuron, a separate gain for each Tm neuron and the offset parameter for the response output nonlinearity.

**Extended Data Fig. 7:**
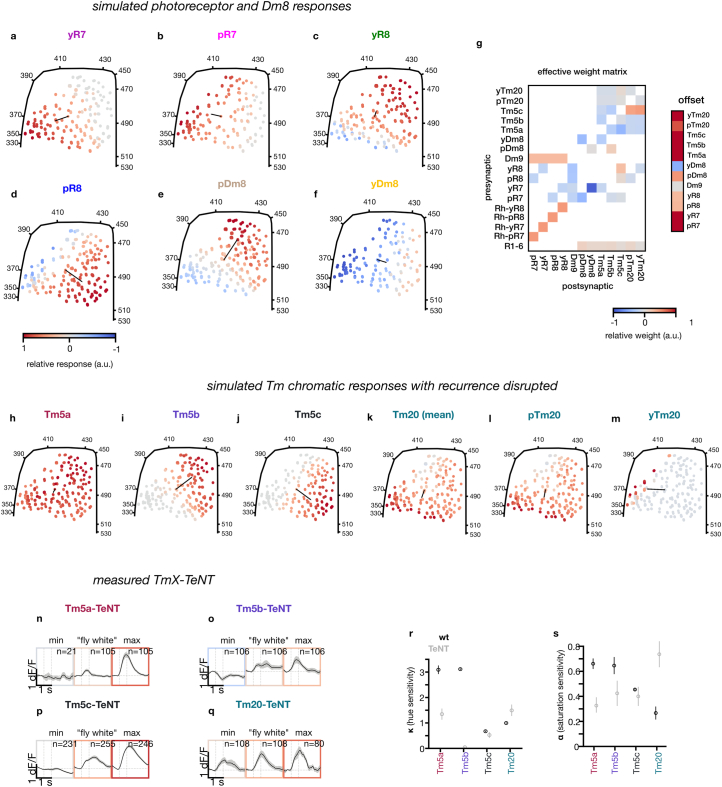
Predicted responses and weight matrix of the fitted circuit model, and response properties of the TmX-TeNT flies. **(a-f)** Predicted responses of photoreceptors and Dm8 neurons in the connectomics constrained circuit model. **(g)** Effective weight matrix (weight matrix multiplied by gain of each neurons) and offset parameters for the circuit model. The Rh-xRx signature indicates the calculated photoreceptor excitations of R7s and R8s at the level of the rhabdomere. pR7, pR8, yR7, and yR8 indicate the axonal segment of the photoreceptors, which we model as a separate node from the rhabdomere. All other neurons are modelled as a single node. **(h-m)** Predicted responses of Tms when silencing Tm recurrence in the circuit model. **(n-q)** Min, ‘fly white’, and max responses of TmX-TeNT flies in the chromatic hyperplane as in Extended Data Fig. 1. **(r)** Mean *κ* values from fitting the nonlinear selectivity model to the wild type and TmX-TeNT responses. *κ* values are obtained using all the data. The error bars indicate the 95% confidence interval of *κ* after fitting the nonlinear selectivity model to the bootstrapped distribution of mean responses. **(s)** Mean *α* values from fitting the nonlinear selectivity model to the wild type and TmX-TeNT responses. *α* values are obtained using all the data. The error bars indicate the 95% confidence interval of *α* after fitting the nonlinear selectivity model to the bootstrapped distribution of mean responses. Source data

The connectome-constrained circuit model fits the measured responses of R7s, R8s, Dm8s and Tm5c at roughly the same level as the LNL model, and Tm5a, Tm5b and Tm20 close to the nonlinear selectivity model (Fig. 4b), despite having fewer parameters. Across the color gamut, simulated responses from our circuit model are strikingly similar to the measured responses (compare Fig. 4f–i with Fig. 2e–h, Extended Data Fig. 7a–d with Fig. 2a–d and Extended Data Fig. 7e,f with Extended Data Fig. 6c,d). The *κ* and *α* values computed by fitting the simulated responses of Tm5a, Tm5b, Tm5c and Tm20 to the nonlinear selectivity model gave values comparable to those obtained directly from the data (Fig. 4c,d). Interestingly, including two types of Tm20s in the model produced *κ* values comparable to those of Tm5a and Tm5b, supporting the idea that our measured Tm20 data are a combination of response from two subtypes with tighter hue selectivity values than their sum (Fig. 4j,k).

As a control, we replaced the weights in our model with random weights, drawn from a uniform distribution (Methods), for the inputs to Tm neurons. We did this 10,000 times and created a null distribution of *R*^2^ values (Fig. 4e). We found that using the connectome synaptic counts for our weights results in significantly better performance than using random weights.

The unexpected prevalence of recurrence in the circuit hinted at an important role for these connections to establish the nonlinear responses of Tm5a, Tm5b and Tm20. We tested this hypothesis in the model, first by removing all recurrent connections between Tm neurons. We did this by setting the direct and indirect weights of the connections between all Tm neurons, including Tm5c, to zero. In this configuration, the circuit model no longer predicts Tm5a, Tm5b and Tm20 responses well, although Tm5c responses are still successfully fit (Fig. 4b). Without recurrence, the tuning of Tm5a, Tm5b and Tm20 became broader (Extended Data Fig. 7h,i,k), while the responses of Tm5c did not change (Extended Data Fig. 7j). Thus, recurrence between Tm neurons is critical for hue selectivity in the model. We thus hypothesized that recurrent connections are essential to tune and sharpen the responses of Tm neurons in vivo.

We cannot disrupt all recurrence at the level of the Tm neurons experimentally. We can however partially disrupt recurrence by blocking the output (but not the response) of single Tm neuron types in vivo, while imaging from the same neuron. We performed this experiment by expressing both GCaMP6f and tetanus neurotoxin (TeNT) in each Tm neuron (Fig. 4l–o and Extended Data Fig. 7n–q), which blocks neurotransmitter release^35^, along with the equivalent manipulation in the model (Fig. 4p–s). When expressing TeNT in Tm5a or Tm5b or Tm20, we found that their responses became broader (Fig. 4l,m,o) and were well fit by a linear model, with the LNL or nonlinear selectivity model not improving the fits (Fig. 4t). Consistently, the hue sensitivity indices (kappa values) derived from the selectivity model were closer to 0 (Extended Data Fig. 7r,s). In the circuit model, when Tm5a or Tm5b or Tm20 outputs were set to 0, the corresponding Tm neuron’s responses similarly became broader (Fig. 4p,q,s), and the circuit model fit these data relatively well (Fig. 4t). In contrast, expressing TeNT in Tm5c did not change the responses pattern of this neuron (Fig. 4n). We did notice some changes in the dynamics and waveform of the responses (Extended Data Fig. 7p), suggesting that the genetic manipulation was effective, but it did not affect tuning, as in the model (Fig. 4r).

In summary, our connectomics-constrained model combined with genetic manipulation of the circuit reveals that the circuit does not require nonlinear synaptic integration (as opposed to an output nonlinearity) to achieve hue selectivity, but rather recurrent connectivity between neurons is necessary for establishing nonlinear hue-selective responses.

## Discussion

Color-related signals have been measured across species that use chromatic information to drive their behaviors. Neurons with narrow spectral tuning have been recorded in various species^36,37^, but hue-selective neurons have only been carefully characterized in the cortex of primates^7–12^. Here we have identified and described the properties of neurons in the optic lobe of the fruit fly that have the characteristics of hue-selective neurons. This finding provided a unique opportunity to define neural circuit mechanisms for the emergence of hue-selective signals in visual circuits in a genetically tractable organism. Using a connectomics-constrained modeling approach, combined with genetic manipulations of the circuit, we showed that recurrent connections are critical for establishing hue-selective signals, without any need for nonlinear synaptic integration. This result highlights recurrence as a fundamental mechanism in biological circuits that enables nonlinear computations to be performed without requiring nonlinear synaptic input integration^8,38–40^. Our findings reveal the circuit basis for a transition from physical detection to perceptual representation in color vision.

Although animals such as birds have been shown to discriminate nonspectral colors behaviorally^26,41^, nonspectral color signals have not been measured explicitly across the animal kingdom. For the fruit fly, it is unclear what the color selectivities we report here correspond to in its visual experience. Many physiological or behavioral studies of color vision in fruit flies have focused on a very narrow part of the fly color space, consisting of blue and green spectral wavelengths^5,42^. This might explain why color behavior in fruit flies has been notoriously difficult to assess. Our measurements suggest that, moving forward, it will be necessary to expand the range of chromatic stimuli to include violet and nonspectral colors, to uncover the role of chromatic information in driving *Drosophila* behavior. Moreover, the tunings we measure here are all in response to full-field stimulus presentations. Future experiments will be necessary to assess how selective their response is for spatial structure, as is well documented in primates^43,44^, and how this relates to their natural environment.

In trichromatic primates, hue-selective neurons have selectivity properties that tile color space. In theory, the range of hues that can be encoded in a tetrachromatic animal such as *Drosophila* is larger than in a trichromatic animal, with two hue angles instead of one, defining a sphere rather than a circle. Although fruit flies do appear to compute a 4D color space, the sphere of hues, at the Tm neuron level at least, is subdivided into only a handful of hue-selective signals (three, or four if we consider two Tm20 subtypes, receiving inputs from either yR8 or pR8). These signals may further be combined downstream of the Tm neurons to give rise to additional hue-selective signals. Alternatively, the fly color vision system may use specific hue-selective signals of particular ethological relevance, rather than provide a general color representation. This system appears to constitute a hybrid between trichromatic primates and an animal such as mantis shrimp, where an unusual number of narrowly tuned photoreceptors function independently, without any convergence of their signals, for specific behavioral programs^45^.

Finally, the emergence of hue signals within the chromatic circuits we studied represents an unexpected degree of processing at such an early stage of visual processing. Traditionally, Tm neurons have been compared to retinal ganglion cells, due to their anatomical position within their respective circuits^14^. However, our empirical findings suggest that, at least in chromatic circuits, Tm neurons perform a function analogous to cortical neurons despite being only one to two synapses downstream of photoreceptors. Mechanistically, the extensive recurrence observed within the medulla is instrumental in producing these nonlinear signals within a relatively shallow/small circuit. In primates, hue selectivity first appears in the primary visual cortex^7,8,10^. Recurrent horizontal connections within the primary visual cortex are prevalent and have been implicated in a variety of functions, including the sharpening and contrast invariance of orientation selectivity^46,47^. The convergent evolution of algorithmic-level computations between vertebrate and invertebrate visual circuits is well documented^14,15^, and already apparent in peripheral color circuits^2^. Thus, we hypothesize that the recurrent mechanism we identified may serve as a foundational basis for hue selectivity across animal brains, including primates. Our findings open up new avenues for further investigation in both invertebrate and vertebrate vision.

## Methods

### Fly genetics

Male and female *w*+ flies were reared on standard molasses-based medium at 25 °C. Rhodopsin Gal4 drivers were used for imaging photoreceptors Rh3-Gal4 and Rh6-Gal^50^ along with Rh4-Gal4 and Rh5-Gal4 (ref. ^51^). Dm8 cells were targeted for imaging using *OrtC2b-Gal4,DIPγ*-*Gal80* (ref. ^31^), and *OrtC1-3-Vp16.AD*; *DIPγ*-*Gal4.DBD*^31^. Tm cells were targeted using the following drivers: Tm5a: *27E03-p65.AD attP40*; *94H07-Gal4.DBD attP2 (SS0788)* (Extended Data Fig. 9); Tm5b: *OrtC1a.DBD;ET24gdVp16.AD*^6^; Tm5c: *OrtC1a-Gal4.DBD*, *OK371-Vp16.AD*^6^; Tm20: *41E03-p65.AD attP40;81G11-Gal4.DBD attP2 (SS00355)*^34^ and *OrtC1a-Gal4.DBD, VP16.AD.tou-9A30* (ref. ^52^).

These lines are specific to the neurons detailed above in the optic lobe, except for the Tm5a and Tm5b lines, which both show some expression in a small population of other neurons. The Tm5a line also labels few L3s in the lamina. The Tm5b line, as described previously, also labels some Tm5a cells^6^. In our hands, of all imaged ROIs in the lobula, only 5% of them were excluded by clustering of the responses, so we estimate the proportion of Tm5a labeled by the Gal4 line to be small. In both cases, this minor nonspecific expression does not affect imaging. In the circuit manipulations, the expression of TeNT in few L3s or Tm5as does not change our interpretation of the results.

Synaptic transmission was blocked using a UAS-TeNT construct *PUAS-TeTxLC.tntG2* (Bloomington Drosophila Stock Center (BDSC), 28838). For stochastic multicolor flpout (MCFO) labeling, a*10XUAS(FRT)myr::smGdP-V5/FLAG/HA-10XUAS(FRT)* construct was used (BDSC, 64087)^53^.

All constructs were expressed heterozygously along with 20X-UAS-GCaMP6f, also expressed heterozygously (BDSC, 42747 and 52869). The genotypes of flies used in each figure are detailed in Supplementary Table 23.

### Two-photon calcium imaging

Recordings were performed as previously described in Heath et al.^2^. Imaging was conducted with a two-photon microscope (Bruker) controlled by PrairieView 5.4 and a mode-locked, dispersion compensated laser (Spectraphysics) tuned to 930 nm. We imaged with a ×20 water-immersion objective (Olympus XLUMPLFLN, 1.0 numerical aperture). In front of the photomultiplier tube (Hamamatsu GaAsP), we mounted a band-pass filter (Semrock 514/30 nm, BrightLine) to reduce bleed-through from the visual stimulus setup. T-series were acquired at 15–30 Hz and lasted for a maximum of 10 min with each frame at *x*–*y* imaging being 160 × 60 pixels (0.58 μm per pixel).

All experimental animals for functional imaging were briefly anesthetized using carbon dioxide on the day of eclosion, and imaged at ages ranging from 3 to 13 days. Flies were prepared for two-photon imaging based on methods previously described^2^. Flies were anesthetized using ice, and mounted in a custom stainless-steel/3D-printed holder. A window was cut in the cuticle on the caudal side of the head to expose the medulla and lobula. Photoreceptors and distal medulla interneurons were imaged in the medulla, whereas Tm axons were imaged in the lobula. The eyes of the fly remained face down under the holder, and remained dry while viewing the visual stimuli, while the upper part of the preparation was covered with saline. The saline composition was as follows: 103 mM NaCl, 3 mM KCl, 5 mM n − tri(hydroxymethyl) methyl − 1Aminoethane − sulfonic acid, 8 mM trehalose, 10 mM glucose, 26 mM NaHCO_3_, 1 mM NaH_2_PO_4_, 1.5 mM CaCl_2_ and 4 mM MgCl_2_, adjusted to 270 mOsm. The pH of the saline was equilibrated near 7.3 when bubbled with 95% O_2_/5% CO_2_ and perfused continuously over the preparation at 2 ml min^−1^. The imaging ROI was limited to the region of the medulla and lobula, neurons are directly activated by stimuli. Specifically, the *z*-depth was zeroed at the same level for each fly (the dorsal part of the lobula) and neural responses were measured from 50–110 microns for the medulla and from 50–90 microns for the lobula below that point. Responses were measured from the rostral fourth of the medulla in that plane. The dorsal third of the eye was covered with black acrylic paint to avoid the region where Rh3 and Rh4 are coexpressed in R7s^54^. Calcium responses were stable throughout imaging. For each fly, we imaged neurons across 5–15 imaging planes (that is, each fly contains around 5–15 recording sessions). No statistical methods were used to predetermine sample sizes, but our sample sizes are similar to those reported in previous publications^2^.

### Visual stimulation

#### Hardware

We produced full-field wavelength-specific stimuli using a customized setup (Fig. 1e). The setup consists of six LEDs in the UV and visible wavelength range (ThorLabs M340L4 - dUV/340 nm; M365L2 - UV/360 nm; M415L4 - violet/415 nm; M455L3 - blue/455 nm; M565L3 - lime/565 nm; M617L3 - orange/615 nm). A customized driver drove the five LEDs from dUV to lime. These LEDs turned on during the return period of the *x*-scanning mirror in the two-photon microscope (fly-back stimulation). We used the TTL signal generated by the two-photon microscope at the beginning of each line-scan of the horizontal scanning mirror (*x*-mirror) to trigger the LED driver. An individual T-Cube (Thorlabs LEDD1B T-Cube) drove the orange LED. Stimuli were generated using customized software written in Python. The update rate for the LED voltage values was 180 Hz.

The different light sources were focused with an aspheric condenser lens (ThorLabs ACL2520U-A) and aligned using dichroic mirrors (dUV-UV dichroic - Semrock LPD01-355RU; UV-violet dichroic - Semrock FF414-Di01; violet-blue dichroic - Semrock Di02-R442; blue-lime dichroic - Semrock FF495-Di03; lime-orange dichroic - Semrock FF605-Di02). The collimated light passed through a diffuser (ThorLabs DG10-1500A) before reaching the eye of the fly, which is positioned 2 cm away.

#### Intensity calibration

In order to measure the intensity of our LEDs across many voltage outputs, we used a photo-spectrometer (250–1,000 nm, Ocean Optics) that was coupled by an optic fiber and a cosine corrector and was controlled using our customized Python software. The photo-spectrometer was mounted on a 3D-printed holder that was designed to fit on our experimental rig and approximately aligned with the fly’s point of view. For each LED, we tested a total of 20 voltage values (linearly separated) from the minimum voltage output to the maximum voltage output. For each voltage value tested, we adjusted the integration time to fit the LED intensity measured, and averaged over 20 reads to remove shot noise.

Using the spectrometer output, we calculated the absolute irradiance (*I*_*p*_(*λ*); in W/m^2^/nm) across wavelengths using equation (1):

1 $${I}_{\rm{p}}(\lambda )={C}_{p}(\lambda )\frac{{S}_{\rm{p}}(\lambda )-{D}_{\rm{p}}(\lambda )}{\Delta t\cdot A\cdot 100}$$

where *C*_p_(*λ*) is the calibration data provided by Ocean Optics (μJ/count), *S*_p_(*λ*) is the sample spectrum (counts), *D*_p_(*λ*) is the dark spectrum (counts), Δ*t* is the integration time (*s*) and *A* is the collection area (cm^2^).

Next, we converted absolute irradiance to photon flux (*E*_q_; in μE/nm) using equation (2):

2 $${E}_{\rm{p}}(\lambda )=\frac{{I}_{\rm{p}}(\lambda )\cdot \lambda }{c\cdot h\cdot {N}_{\rm{A}}\cdot 1{0}^{6}}$$

where $\frac{h\cdot c}{\lambda }$ is the energy of a photon with *h* as Planck’s constant (6.63 × 10^−34^ *J* ⋅ *s*), *c* as the speed of light (2.998 ⋅ 10^8^ *m*/*s*), and *λ* the wavelength (nm). *N*_A_ is Avogadro’s number (6.022 × 10^23^ mol^−1^).

The minimum intensity is zero for all LEDs, and the maximum intensities are: dUV ≈ 6 μE, UV ≈ 7 μE, violet ≈ 11 μE, blue ≈ 18 μE, lime ≈ 25 μE and orange ≈ 160 μE.

#### Stimulus design

Each stimulation protocol had at least 15 s before and after the stimulation period in order to measure baseline fluorescence (fluorescence to background light). Because we wanted to replicate natural spectral distributions of light, we adapted the eye to a combination of LEDs that mimic natural light conditions at dawn. We chose dawn-like conditions, because this is when flies are most active. We fit the LED intensities to the background light condition using methods described in Christenson et al.^22^. Our background LED intensities were: 0.01 μE for dUV, 0.06 μE for for UV, 0.1 μE for violet, 0.25 μE for blue, 0.33 μE for green and 0.25 μE for orange. Flies were adapted to the background light for approximately 5 min before the start of the recording sessions. The background light was maintained between individual recordings.

During the stimulation period, individual stimuli were randomly interleaved. Each stimulus was a step stimulus that was 0.5 s long. The interstimulus interval was 1.5 s or 2 s long.

**Contrasts stimulus set:** For the contrast stimulus set, we flashed the LEDs individually and selected mixtures of LEDs. The intensity steps were added on top of the chosen background light with added intensities of 0.1 μE, 0.3 μE, 0.5 μE, 0.75 μE, 1 μE and 3 μE. For LED mixtures, the intensity of each individual LED was set to these additive intensity steps, so that for example a mixture of violet and blue at 1 μE of added intensity has a total intensity of 2 μE plus the total background intensity.

The LED mixtures sampled were (using the single letter annotations for the LEDs): D + U, U + L, D + U + L + O, V + B + L + O, U + V + B, B + L + O, D + L + O, D + U + V + B + L + O, V + B, D + U + O, and D + U + V. Within each imaging session, we flashed all stimuli in the set once.

**Extended Data Fig. 8:**
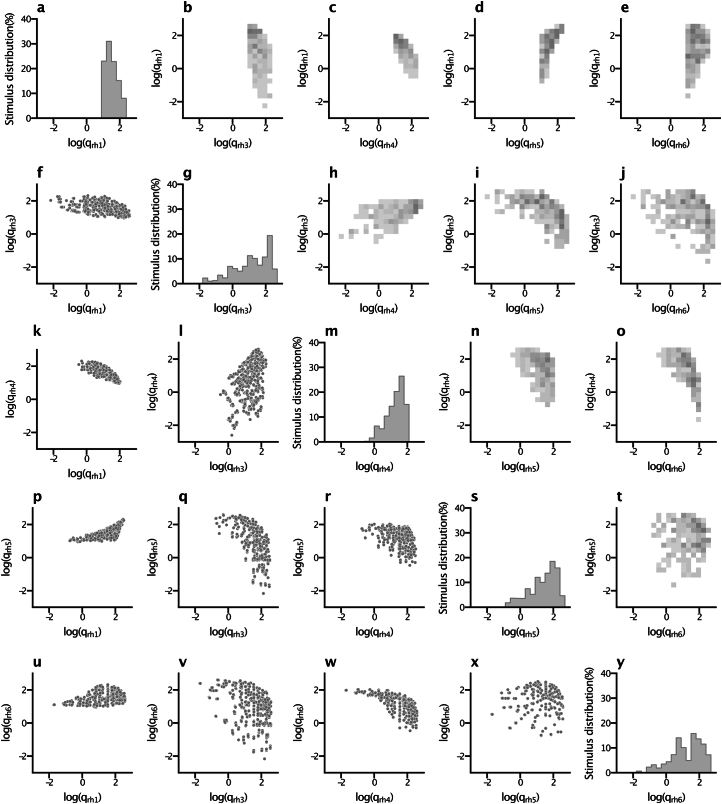
Distribution of relative capture values in the color gamut stimulus set. **(a-y)** Distribution of calculated relative captures for the Rh1, Rh3, Rh4, Rh5, Rh6 opsin. The diagonal panels show the histogram for each opsin as a percentage of the stimulus set. The panels in the lower triangle are scatter plots of the individual stimuli for pairwise comparisons between opsins. The panels in the upper triangle are 2D-histograms of paired opsins.

**Color gamut set:** For the color gamut set, we flashed different mixtures of LEDs. We obtained the LED intensities by first randomly sampling capture values in the fly color space around an overall relative capture of 5 (Extended Data Fig. 8), and then fitting the target captures using methods previously described in Christenson et al.^22^. For each imaging session, we flashed a subset of this stimulus set (approximately 20% of all stimuli) and repeated each stimulus three times. We implemented a random subsampling method that ensured we span color space for each imaging session. To do this, we first randomly selected a stimulus in color space. Then, we iteratively sampled from a subset of ten not-yet-chosen stimuli that are maximally distant from the already sampled stimuli. The convex hull of the color gamut stimulus set covers approximately 90% of a set of natural reflectances within the fly’s chromatic hyperplane^55^, thus allowing for a fairly complete characterization of a neuron’s chromatic tuning properties across the set of possible fly colors.

### Quantification of imaging data

All data analysis for in vivo calcium imaging was performed in Python 3.8 using custom-made Python code and publicly available libraries. First, we removed minor remaining bleed-through artifacts from our LED system by subtracting the 10th percentile value of each column of pixels in each image. To correct our calcium movies for motion, we performed rigid translations based on template alignment using the algorithm provided by the CaImAn package^56^.

#### Image denoising

To denoise our calcium movies, we implemented a version of Kernel principal component analysis (PCA) that has been shown to reduce different types of noise in images^57,58^. First, we reshaped the movie so that all rows correspond to a frame and all columns correspond to the flattened image (*n*_frames_ × *m*_pixels_). Then, we concatenated seven 7 *n*-frame-shifted versions of the movie horizontally $\left({n}_{\rm{frames-7}}\times \left({m}_{\rm{pixels}}\times 7\right)\right)$. We performed Kernel PCA on this final data matrix using 512 components and a radial basis function. The hyperparameters of the Kernel PCA model were set to the standard values of the sklearn.decomposition.KernelPCA class function. We obtained the denoised version of the movie by taking the last *m*_pixels_ columns of the reconstructed data matrix and reshaping it back into the movie format.

#### Image segmentation

ROIs were selected automatically using a custom-made approach and verified manually. A local correlation projection was taken of the complete motion-corrected and denoised image stack. We thresholded the projected image in three ways to identify pixels that are certainly part of a ROI (upper threshold), certainly part of the background (lower threshold) and possibly part of a ROI (medium threshold). The thresholds were chosen by fitting a two-component Gaussian Mixture model to the pixel values of the projected image using the sklearn.mixture.GaussianMixture class function. The lower and upper threshold was set to the estimated mean of the component with a lower and higher value, respectively. The medium threshold was set to the weighted average of the two means, weighted by the variance of each component. The thresholded images were used to identify connected components (that is, individual ROIs). Next, we applied a watershed transformation to obtain the individual ROIs. We discarded any ROIs of fewer than four pixels.

#### Signal extraction

To extract calcium traces from our segmented images, we first took the average fluorescence of each ROI at each time point. We subtracted the mean background fluorescence—the mean fluorescence of all pixels that do not belong to any ROI—from each trace to remove background fluctuations. To calculate the d*F/F* signal, we use as a baseline for our denoised traces the 25th percentile of a rolling 40-s time window. Finally, we smooth our d*F/F* signal with a Savgol filter of 0.5 s in size and a third-order polynomial. We discarded ROIs, where the signal-to-noise (SNR) ratio was smaller than 2. The SNR was defined as the magnitude of the amplitude responses during stimulation over the magnitude of the baseline responses before and after the start of stimulation (SNR = ∥*v*_stim_∥^2^/∥*v*_baseline_∥^2^). Amplitude responses during stimulation were calculated by taking the mean d*F/F* signal between the 0.35 and 0.5 second during stimulation and subtracting the mean value between the 0.35 and 0.1 second before stimulation. Baseline responses were calculated by randomly taking mean values of a 0.15-s duration before and after the start of the stimulation protocol.

#### Response averaging and normalization

After removing noisy ROIs, we averaged the individual stimulus-aligned traces. Next, we calculated our final estimate of the amplitude using these averaged stimulus-aligned traces. As before, we took the mean d*F/F* signal between the 0.35 and 0.5 second during stimulation and subtracted the mean value between the 0.35 and 0.1 second before stimulation. Next, we obtain a normalized d*F/F* response vector ${{{{\bf{v}}}}}^{{\prime} }$ for each neuron by dividing the unnormalized response vector (**v**) by its estimated standard deviation from zero according to equation (3):

3 $${{{{\bf{v}}}}}^{{\prime} }=\frac{{{{\bf{v}}}}}{\sqrt{\frac{1}{N-1}{\sum }_{\rm{i}}{v}_{\rm{i}}^{2}}}$$

where *v*_i_ is the unnormalized response of a neuron to stimulus *i*, and *N* is the total number of stimuli. In all our plots where we indicate d*F/F* amplitudes, we show this normalized d*F/F* signal. For the tetrahedral plots, we show maximum-normalized responses to ensure equal scaling of the colored response map across neurons.

Data collection and analysis were not performed blind to the conditions of the experiments.

### Modeling

The stimuli we used are characterized by computed photon activations for the four fly opsins, labeled by *μ* = 1, 2, 3, 4, and given by ${X}_{\rm{\mu} }=\log (({q}_{\rm{\mu} }+0.001)/1.001)$, with *q*_*μ*_ the calculated relative capture for opsin *μ*, as described previously in Heath et al.^2,22^. We decompose the components of the 4D vector **X** into a 3D vector $\overrightarrow{\mathbf{x}}$ lying within the color tetrahedron and a scalar *l* that is the projection of **X** along the axis connecting the zero point in the full color space to the white point in the color tetrahedron. The color of each stimulus in all our models is then described by the 3D vector $\overrightarrow{\mathbf{x}}$, which connects the white point at the center of the color tetrahedron to the projection of the color point of the stimulus into the tetrahedron. We also define $\hat{\mathbf{x}}$ as the vector $\overrightarrow{\mathbf{x}}$ normalized to unit length. The stimuli were designed to be of equal luminance but, because this could not be achieved exactly, we included a term in our models proportionate to the luminance *l* of each stimulus.

Each neuron is characterized by a 3D color preference ${\hat{\mathbf{p}}}$, which is a vector of unit length in the color tetrahedron, an overall amplitude factor *a*, and the coefficient multiplying the luminance *l*, denoted by *b*. This is the full complement of parameters for the linear model, whereas there are additional parameters in the other models, as described below. Note that ${\hat{\mathbf{p}}}\cdot {\hat{\mathbf{x}}}$ can be expressed as $\cos (\theta )$, with *θ* the angle between ${\hat{\mathbf{p}}}$ and ${\hat{\mathbf{x}}}$. Similarly, $\hat{\mathbf{p}}\cdot \overrightarrow{\mathbf{x}}=s\cos (\theta )$, with $s=| \overrightarrow{\mathbf{x}}|$ the saturation. The input for stimulus *i* is characterized by ${\overrightarrow{\mathbf{x}}}_{\rm{i}}$ and *l*_*i*_, and this generates a model response *y*_*i*_. The corresponding response from the data is *v*_i_. All fits were done by minimizing the squared difference between *y*_*i*_ and *v*_*i*_, summed over *i*, except for the circuit model for which we maximized the sum of the correlation coefficients of *y*_i_ and *v*_i_ for reasons given below.

#### Linear model

In the linear model, the predicted response to stimulus *i* is given in equation (4):

4 $${y}_{\rm{i}}={a}_{{{\mbox{L}}}}\hat{\mathbf{p}}\cdot {\overrightarrow{\mathbf{x}}}_{\rm{i}}+b{l}_{\rm{i}}={a}_{{{\mbox{L}}}}{s}_{\rm{i}}\cos ({\theta }_{\rm{i}})+b{l}_{\rm{i}}.$$

The model has four parameters: *a*_L_, *b* and two parameters that specify $\hat{\mathbf{p}}$.

#### LNL model

For the LNL model, we first computed a linear response ${\tilde{y}}_{\rm{i}}$ using equation (4) and then passed this through an output nonlinearity given by a modified tanh function^59^, according to equation (5):

5 $${y}_{\rm{i}}=\left\{\begin{array}{ll}{a}_{{{\mbox{NL}}}}(1+\gamma )\tanh \left(\frac{{\tilde{y}}_{\rm{i}}}{1+\gamma }\right)\quad &\,{{\mbox{for}}}\,\,{\tilde{y}}_{\rm{i}}\le 0\\ {a}_{{{\mbox{NL}}}}(1-\gamma )\tanh \left(\frac{{\tilde{y}}_{\rm{i}}}{1-\gamma }\right)\quad &\,{{\mbox{for}}}\,\,{\tilde{y}}_{\rm{i}} > 0.\end{array}\right.$$

The additional parameters, beyond the four of the linear model, *a*_NL_ and *γ* were determined using the nonlinear least-squares method (scipy.optimize.least_squares).

#### Nonlinear selectivity model

The nonlinear selectivity model uses the parameters *a*_NL_, *b* and $\hat{\mathbf{p}}$ and adds two new parameters, *κ* (hue sensitivity) and *α* (saturation sensitivity), according to equation (6):

6 $${y}_{\rm{i}}=\frac{{a}_{{{\mbox{NL}}}}| \overrightarrow{\;{\mathbf{x}}_{\rm{i}}}{| }^{\alpha }}{\kappa }\left(\exp \left(\kappa \hat{\mathbf{p}}\cdot \hat{{\mathbf{x}}_{\rm{i}}}\right)-1\right)+b{l}_{\rm{i}}=\frac{{a}_{{{\mbox{NL}}}}{s}^{\alpha }}{\kappa }\left(\exp \left(\kappa \cos ({\theta }_{\rm{i}})\right)-1\right)+b{l}_{\rm{i}}$$

To fit this model, we optimized the parameters $\hat{\mathbf{p}},{a}_{{{\mbox{NL}}}}$, and *b* over a grid of values for *κ* and *α*, and determined the best fit across the grid. The *κ* values varied between 10^−2^ and 10^1^ in 20 uniform log steps, and *α* values varied between 10^−1^ and 10^1^ in 13 uniform log steps.

#### Model fitting

For all models, we used the color gamut data in our fitting procedure. Because this dataset has a different number of observations for different stimuli (see color gamut set stimulus details in ‘Stimulus design’), we weighted each color point during training by the number of observations. To assess goodness of fit, we calculated noise-corrected *R*^2^ values according to ref. ^60^, as given by equation (7),

7 $${R}^{2}=\frac{{\sum }_{i}{m}_{i}{\left(\;{y}_{i}{v}_{i}\right)}^{2}-\frac{{\sigma }^{2}}{\overline{m}}{\sum }_{i}{m}_{i}{y}_{i}^{2}}{{\sum }_{i}{m}_{i}{y}_{i}^{2}{\sum }_{i}{m}_{i}{v}_{i}^{2}-\frac{{\sigma }^{2}\left(\hat{m}-1\right)}{\overline{m}}{\sum }_{i}{m}_{i}{y}_{i}^{2}}$$

where *m*_*i*_ is the number of observations for stimulus $i,\overline{m}$ is the average number of observations across stimuli, $\hat{m}$ is the total number of observations across stimuli, and *σ*^2^ is the estimated noise as described in the signal extraction section (that is, *σ*^2 ^= ∥***v***_baseline_∥^2^).

#### Circuit model

The circuit model is a recurrent network constrained by the direct and indirect connections of the EM-reconstructed medulla neuropil connectome. The prediction of the model for *a* = 1, 2, …, 12, representing pR7, yR7, pR8, yR8, Dm9, pDm8, yDm8, Tm5a, Tm5b, Tm5c, pTm20, yTm20, respectively, is denoted by *y*_*a*_. These responses are given by a system of ordinary differential equations with time constants *τ*_*a*_, as shown in equation (8),

8 $${\tau }_{a}\frac{d{y}_{a}}{dt}=-{y}_{a}+f\left({g}_{a}\left(\mathop{\sum }\limits_{b=1}^{12}{w}_{ab}{y}_{b}+\mathop{\sum }\limits_{\mu =1}^{4}{j}_{a\mu }{X}_{\mu }+{p}_{a}{x}_{0}\right)\right).$$

This equation was transformed into a fixed point problem by setting the left-hand side to zero. This allowed us to model the data as being measured at steady state, consistent with the differential equation at equilibrium. To solve the resulting fixed point problem, we utilized Anderson acceleration (AA) as described in ref. ^61^. AA is an iterative method that uses a fixed number from a prior iterate to extrapolate and accelerate convergence to a new fixed point. The AA solver was run until convergence below a set tolerance (<1 × 10^−4^), providing a steady-state solution to the differential equation that was then used to fit the model parameters to the amplitude data. We used a form of implicit differentiation instead of directly backpropagating through AA in order to speed up and achieve stable training^62^. With this approach, we sped up the time to optimize the model parameters. We applied this optimization procedure for all circuit-constrained models.

The function *f* is the modified tanh described in equation (5) with *a*_NL_ = 1 and with *γ*_*a*_ a free parameter for each neuron, except for Dm9, which used the fixed value *γ* = 0. Thus, a total of 11 free parameters determine the *γ* values. In addition, *g*_*a*_ is the gain parameter for neuron *a*, *j*_*aμ*_ are input weights for the computed opsin activations^2^, and *w*_*ab*_ is the weight of the connection from neuron *b* to neuron *a*. All weights to a neuron were normalized, so that their absolute value summed up to 1 (1 = ∑_*b*_∣*w*_*ab*_∣ + ∑_*μ*_*j*_*aμ*_). We also included the term *p*_*a*_*x*_0_, where *x*_0_ is the non-chromatic input from the R1–6 photoreceptors, and *p*_*a*_ is a neuron-specific weight for that input. We assigned equal gains to the two Dm8 neurons and for all four photoreceptors, so there are a total of eight parameters characterizing the 12 neuronal gains of the model. Only four of the neurons have nonzero matrices *j*_*aμ*_, because opsin activations only affect photoreceptor neurons directly. The remaining 4 × 4 matrix is diagonal, and we used the same value for all four photoreceptors^2^. Thus, the full 12 × 4 matrix *j*_*aμ*_ is characterized by a single parameter. There are only seven nonzero values of *p* because R1–6 only synapses onto the two Dm8s and the five Tm neurons. The 12 × 12 matrix **w** (Extended Data Fig. 7g) is almost entirely taken from our connectome synapse counts and from Heath et al.^2^. To obtain the weights of Tm recurrence, we added both the monosynaptic and the disynaptic connections between Tm neurons. The only exceptions to a full determination of **w** are the connections between the two Dm8 neurons, which were not reconstructed and are thus determined by two additional parameters. All told, the total number of parameters is 29.

The weights from Dm8s, R7s and R8s to Tm neurons as well as the recurrent weights between Tm neurons are fixed according to their proportional input, as obtained from our EM reconstruction. We constrained R1–6 inputs to Dm8 to be positive and R7 inputs and indirect connections to Dm8 to be negative, as shown previously^28,29^. The weights and signs of the R1–6 inputs were varied freely. We set R7s and R8s to be inhibitory onto Tm5a, Tm5b and Tm20 and excitatory for R8s onto Tm5c, as R8s can be both inhibitory (via histamine transmission) and excitatory (via acetylcholine transmission)^34^. The signs of Dm8 inputs onto Tm neurons were positive for Tm5b, and negative for yDm8 onto Tm5a. For the recurrent weights between Tm neurons, we included both indirect and direct connections between Tm neurons to calculate the proportional input to each Tm neuron for each other Tm neuron. For the effective signs of the recurrent weights, we tested models with different signs for each connection and chose the set of signs that resulted in the best fit. To do this, we used the package optuna to effectively iterate different sets of signs^63^.

Fitting was based on the correlation coefficient *r*_*a*_ between the simulated and measured responses for each neuron. We use correlations instead of squared errors, because our calcium indicator only measured the relative amplitudes of responses. We obtained our loss function by summing the negative of the correlation over neurons, according to equation (9),

9 $$L=-\mathop{\sum }\limits_{a=1}^{12}\frac{{\sum }_{i}{m}_{i}{v}_{ia}{\,y}_{ia}}{\sqrt{{\sum }_{i}{m}_{i}{v}_{ia}^{2}}\sqrt{{\sum }_{i}{m}_{i}{\;y}_{ia}^{2}}},$$

where *y*_*ia*_ is the response of neuron *a* to stimulus *i*. We fit the model in three stages, first fitting the parameters for the photoreceptor–Dm9 circuit, then for the Dm8 circuit, and finally the Tm circuit. For each stage, we fit the parameters using backpropagation with PyTorch’s autograd functionality and its Adam optimizer. Each stage was fit using batches of 64 stimuli with a total of 100 epochs. We chose a learning rate of 0.001 and assessed convergence by calculating the loss across all data points after each epoch. We stopped the fitting procedure early if convergence was reached before 100 epochs. To construct the null distribution for the synaptic count constrained model, we randomly sampled weights from a standard uniform distribution and normalized them (as we did for the synaptic count data) for all the inputs to Tm neurons 10,000 times and refit the model using the same procedure. Next, we compared the mean *R*^2^ of Tm neurons for the synaptic count constrained model to this distribution to assess if the model significantly outperformed a random weight model.

#### Interpolation

To interpolate responses between sampled points in photoreceptor excitation space, we implemented a radial basis function (RBF) interpolator using a modified version of the scipy.interpolate.RBFInterpolator class. The modification was simply to remove a bias term that was not needed given that our measured amplitudes are baseline subtracted. For our RBF kernel, we chose the thin plate spline, a common spline-based technique for data interpolation and smoothing^64^. To ensure that we were working in an interpolative regime, we projected all points outside the convex hull of the training set onto the convex hull, using methods previously developed in the drEye Python package introduced in ref. ^22^. We fit the interpolator by combining both the color gamut dataset and the contrast stimulus set into a single dataset. This increased the volume of the convex hull, and thus reduced clipping of interpolated responses.

For the single wavelength interpolation, we first calculated the excitations of each photoreceptor to a set of lights that follow a Gaussian distribution with a width of 10 nm and peaks ranging from 320 nm to 580 nm. We normalized the calculated excitation for each single wavelength so that they had the same constant value as the isoluminant plane chosen for our gamut stimulus experiment. To obtain nonspectral lines, we took the set of single wavelengths and connected the single wavelength stimulus points in color space that correspond to the maximum excitation of each nonadjacent opsin.

For the single wavelength interpolation, we first calculate the excitations of each photoreceptor to a set of lights that follow a Gaussian distribution with a width of 10 nm and peaks ranging from 320 nm to 580 nm. We normalize the calculated excitation for each single wavelength so that they have the same constant value as the isoluminant plane chosen for our gamut stimulus experiment. To obtain nonspectral lines, we took the set of single wavelengths and connected the single wavelengths stimulus points in color space that correspond to the maximum excitation of each nonadjacent opsin.

### Statistical analysis

#### Bootstrapping

We calculated bootstrapped distributions of our estimates, such as the mean response, to assess significance and avoid assuming normality. Any bootstrapped distribution or calculated confidence intervals shown are the result of empirical bootstrapping of independently chosen subsets of ROIs. For each bootstrap sample, we obtained mean responses as for the complete dataset and calculated the various indices and fits as before. We performed a total of 1,000 bootstrap iterations to obtain our empirical distributions.

#### Sparsity index

To obtain the sparsity index, we calculated the absolute responses and normalized by the maximum absolute response and then averaged across these normalized responses, as given by equation (10):

10 $${\mathtt{Index}}=1-\frac{1}{N}\mathop{\sum }\limits_{i=1}^{N}\frac{| {v}_{i}| }{\max \left(| {v}_{1}| ,| {v}_{2}| \ldots | {v}_{N}| \right)}$$

#### Luminance invariance index

To obtain the luminance invariance index, we first performed two types of regressions on the data for each neuron, according to equations (11) and (12):

11 $${y}_{i}=b{l}_{i}$$

12 $${y}_{i}={a}_{{{\mbox{L}}}}\hat{p}\cdot {\overrightarrow{\mathbf{x}}}_{i}.$$

Next, we divided the obtained *R*^2^ value for equation (12) by the obtained *R*^2^ value for equation (11). We defined this ratio as our luminance invariance index.

#### Hue sensitivity index

We quantified the sharpness of neural tuning to hue using a hue sensitivity index that ranges from 0 to 1. The hue sensitivity index measures the alignment between hue directions in the stimulus space and the mean neural responses, weighted by the number of observations. To ensure a spherical distribution of stimuli for reliable measurement of hue sensitivity, we restricted analysis to the 2D opponent space and further to stimuli with saturation of less than 50% of the maximum. This retains responses distributed spherically around the ‘white point’.

To compute the hue sensitivity index, we first normalized the opponent stimulus values **P** to unit length, according to equation (13):

13 $${{{{\bf{P}}}}}_{{{{\rm{normalized}}}}}=\frac{{{{\bf{P}}}}}{\parallel {{{{\bf{p}}}}}_{i}{\parallel }_{2}}$$

We then scaled the saturation values ∥**p**_*i*_∥_2_ to range from 0 to 1, retaining only stimuli below 0.5 saturation. For these stimuli, we computed a hue sensitivity vector **h**_vec_ (similar to the Raleigh vector used in ref. ^9^) as a weighted vector sum of the hue directions **X**_normalized_, using the mean responses *y*_*i*_ and number of observations *m*_*i*_ as weights, according to equation (14):

14 $${{{{\bf{h}}}}}_{{{{\rm{vec}}}}}=\frac{{\sum }_{i}{m}_{i}{{{{\bf{p}}}}}_{{{{\rm{normalized}}}},i}{\,y}_{i}}{{\sum }_{i}{m}_{i}| {\,y}_{i}| }$$

We plot this vector with the data in the 2D color-opponent plots. Finally, the hue sensitivity index (HSI) is the L2 norm of **h**_vec_, according to equation (15):

15 $${\mathrm{HSI}}=\parallel {{{{\bf{h}}}}}_{{{{\rm{vec}}}}}{\parallel }_{2}$$

Values near 1 indicate selective tuning to a single hue direction, while values near 0 indicate equivalent responses across hues.

### EM reconstructions

We used the EM dataset from the female adult fly brain *D. melanogaster* from Zheng et al.^16^ to obtain connectomic information. We chose two skeletons of Tm5a, Tm5b, Tm5c and Tm20 previously reconstructed on CATMAID^3^ and found them on FlyWire^17,18^. We obtained programmatic access to the FlyWire server and used FAFBseg and NAV is Python libraries to visualize all neurons, find all synapse locations (pre/post), list all presynaptic and postsynaptic segments connected to our seed neurons and predict the neurotransmitter identity of each cell^65,66^. The connections between seed Tm neurons and photoreceptors were obtained from previously published analysis^3^ and refined on CATMAID. For all other connections, after listing all the presynaptic and postsynaptic partners to the Tm cells, we reconstructed them in the FlyWire environment, through the web interface flywire.ai, in order to identify their cell class or cell type based on their anatomy. We reconstructed and identified all presynaptic segments with >2 synapses (1,488 sites; Supplementary Table 1) and postsynaptic segments with >4 synapses (1,479 sites; Supplementary Table 2). In the case of Dm8s, we selected ‘seed’ pDm8 and yDm8 neurons among a set of previously identified cells from this volume^3^ and identified them in FlyWire. We reconstructed and identified all presynaptic segments with >2 synapses (pDm8:284; yDm8:487 sites; Supplementary Table 1) and postsynaptic segments with >4 synapses (pDm8:487; yDm8:750 sites; Supplementary Table 2). Home column photoreceptor presynaptic inputs had previously been identified^3^, while photoreceptor inputs to other columns were identified on CATMAID. Altogether, we found that our seed Tm and Dm8 neurons had 2,754 postsynaptic sites and 6,162 presynaptic sites; 2,084 sites (75.67%) represent inputs from 296 presynaptic neurons (>2 synapses), and 2,132 sites (34.6%) are outputs to 270 postsynaptic neurons (>4 synapses; Supplementary Table 2). Detailed connectivity (inputs and outputs) of each individual neuron can be found in Supplementary Tables 3–22.

### Immunohistochemistry

Immunostainings were done following the Janelia FLyLight MCFO immunohistochemistry protocol. Adult flies were anesthetized on ice. Brains were dissected in cold Schneider’s Insect Medium (S2) and fixed in 2% paraformaldehyde diluted in S2 for 60 min at room temperature (RT). To prevent nonspecific binding, brains were incubated in 5% goat serum (GS) blocking solution diluted in 0.5% Triton X-100 in PBS (PBT) for 90 min at RT. Brains were incubated at 4 °C overnight with the following primary antibodies: mouse nc82 (1:30 dilution; DSHB), rabbit α-HA (C29F4; 1:300 dilution; Cell Signaling Technologies) and rat *α*-FLAG (1:200 dilution; Novus Biologicals) in 5% GS diluted in PBT. Secondary antibodies Cy 2 AffiniPure goat *α*-mouse (1:500 dilution; Jackson ImmunoResearch), AF594 donkey *α*-rabbit (1:500 dilution; Jackson ImmunoResearch) and AF647 donkey *α*-rat (1:150 dilution; Jackson ImmunoResearch) were incubated for 4 h at RT in 5% GS diluted in PBT. Images were acquired using an LSM 800 (Zeiss) confocal microscope with an LD LCI Plan-Apochromat ×25/0.8 Imm Corr DIC M27 objective (Zeiss).

### Reporting summary

Further information on research design is available in the Nature Portfolio Reporting Summary linked to this article.

## Online content

Any methods, additional references, Nature Portfolio reporting summaries, source data, extended data, supplementary information, acknowledgements, peer review information; details of author contributions and competing interests; and statements of data and code availability are available at 10.1038/s41593-024-01640-4.

## Supplementary information

Supplementary Information Supplementary Tables 1–23.

Reporting Summary

## Source data

Source Data Fig. 1 Photoreceptor and Tm GCaMP6f raw trace data.

Source Data Fig. 2 Photoreceptor and Tm GCaMP6f gamut response data.

Source Data Fig. 3 Data for modeling.

Source Data Fig. 4 Tm and Tm-TeNT GCaMP6f data.

Source Data Extended Data 1 Photoreceptor and Tm GCaMP6f gamut response data.

Source Data Extended Data 2 R7 GCaMP6f mixture data.

Source Data Extended Data 3 Data for modeling.

Source Data Extended Data 4 Data for presynaptic connectivity.

Source Data Extended Data Fig. 5 Data for recurrent connectivity.

Source Data Extended Data Fig. 6 Dm8 GCaMP6f gamut response data.

Source Data Extended Data Fig. 7 Tm and Tm-TeNT GCaMP6f data.

**Extended Data Fig. 9:**
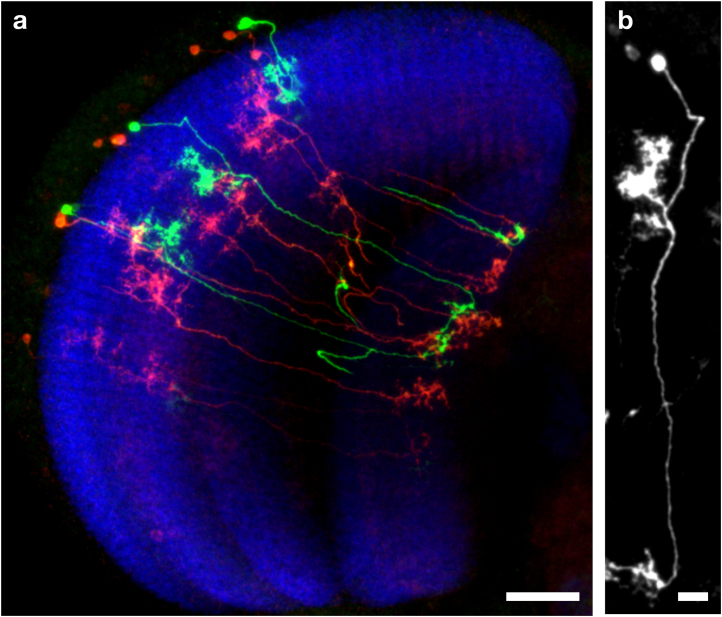
Characterization of the Tm5a split Gal4 line (*27E03-p65.AD; 94H07-Gal4.DBD attP2*). **(a)** MCFO staining with two stop-cassette reporters HA and FLAG. 53 *μ*m section, maximum intensity projection. Scale bar 20 *μ*m. **(b)** Single Tm5a clone. Inset from panel a. Scale bar 5 *μ*m. This is a representative example of 6 brains that were dissected and stained. Source data

Source Data Extended Data Fig. 9 Unprocessed confocal image R27E03AD, R94H07DBD.GCaMP6f, MCFO, FLPG5, PEST.

## Data Availability

The processed and averaged d*F/F* amplitudes for each cell type can be found at https://gitlab.com/rbehnialab/chreyesees. Connectomics data are provided in the Supplementary Information. The raw data are available via Zenodo at 10.5281/zenodo.10720630 (ref. ^67^). Source data are provided with this paper.

## Code availability

Custom analysis code used in the manuscript can be found via GitLab at https://gitlab.com/rbehnialab/chreyesees/.
