# Supplementary material for: Hue selectivity from recurrent circuitry in Drosophila
Source: Nat Neurosci. 2024 May 16;27(6):1137–47. doi: 10.1038/s41593-024-01640-4 (PMC11537989; doi:10.1038/s41593-024-01640-4)
Supplement: Supplementary file 1 — Supplementary Tables 1–23. [file 41593_2024_1640_MOESM1_ESM.pdf]

nature neuroscience

Article https://doi.org/10.1038/s41593-024-01640-4

Hue selectivity from recurrent circuitry in *Drosophila*

In the format provided by the authors and unedited

Flywire ID	cell type	pre sites total (n)	pre sites identified (n)	pre sites identified (%)	neurons (n)
**720575940638634960**	pDm8	284	217	76.4	31
**720575940638424895**	yDm8	462	379	82	47
**720575940639473998**	Tm5a	227	152	67	21
**720575940614902495**	Tm5a	140	107	76.4	17
**720575940627282584**	Tm5b	328	225	68.6	41
**720575940660846721**	Tm5b	249	171	68.7	31
**720575940619252603**	Tm5c	240	150	62.5	30
**720575940627314521**	Tm5c	280	227	81.1	27 (26)
**720575940635252890**	Tm20	274	239	87.2	24 (22)
**720575940628559366**	Tm20	270	217	80.4	27
	Total	2754	2084	75.7	296 (293)

Table 1. Quantification of presynaptic sites to seed Dm8 and Tm cells. (col.1-2) FlyWire segment id and cell type. (col. 3) Total number of presynaptic connections from all presynaptic neurons. (col. 4) Identified presynaptic sites from cells forming >2 synapses with the seed neurons. (col.5) Percentage of identified presynaptic sites vs total presynpatic sites. (col. 6) Total number of presynaptic neurons forming >2 synapses for each seed neuron. Numbers shown inside () reflect the number of neurons identified to a cell class or a cell type. When omitted all neurons have been identified to a cell class or type.

Flywire ID	cell type	post sites total (n)	post sites identified (n)	post sites identified (%)	neurons (n)
**720575940638634960**	pDm8	487	286	58.8	28 (26)
**720575940638424895**	yDm8	750	375	50	64 (63)
**720575940639473998**	Tm5a	496	144	29	16 (15)
**720575940614902495**	Tm5a	386	154	39.9	19 (15)
**720575940627282584**	Tm5b	563	156	27.7	18 (15)
**720575940660846721**	Tm5b	542	158	29.2	21 (19)
**720575940619252603**	Tm5c	908	256	27.9	29 (25)
**720575940627314521**	Tm5c	812	218	25.4	22 (18)
**720575940635252890**	Tm20	620	236	38.1	31 (24)
**720575940628559366**	Tm20	598	172	28.8	26 (23)
	Total	6162	2155	35	274 (243)

Table 2. Quantification of postsynaptic sites to seed Dm8 and Tm cells. (col.1-2) FlyWire segment id and cell type. (col. 3) Total number of postsynaptic connections from all postsynaptic neurons. (col. 4) Identified postsynaptic sites from cells forming >4 synapses with the seed neurons. (col.5) Percentage of identified postsynaptic sites vs total postsynpatic sites. (col. 6) Total number of postsynaptic neurons forming >4 synapses for each seed neuron. Numbers shown inside () reflect the number of neurons identified to a cell class or a cell type.

Flywire ID	cell type	pre sites (n)
**720575940632586297**	pR7	39
**720575940615537314**	pR7	4
**720575940612694165**	yR7	15
**720575940641088640**	yR7	11
**720575940628300090**	yR7	4
**720575940631563515**	pR7	2
**720575940624675079**	pR7	2
**720575940618504395**	Mi1	4
**720575940623904136**	Mi3	6
**720575940628189457**	Dm	3
**720575940622692265**	Dm2	3
**720575940614131119**	Dm5	5

**720575940630969303**	Dm	14
**720575940612167537**	yDm8	3
**720575940655698849**	Dm9	3
**720575940648106116**	Dm	8
**720575940622466036**	CB0566	7
**720575940615181479**	Sm19	18
**720575940633387181**	Sm19	3
**720575940641982709**	Sm20	7
**720575940615314834**	Sm20	7
**720575940605990590**	Sm19	7
**720575940613095282**	Sm20	6
**720575940618262050**	Sm21	3
**720575940622540894**	Sm21	4
**720575940622315647**	Sm20	3
**720575940627733777**	Sm20	6
**720575940633138771**	Sm20	5
**720575940617838225**	Sm26	12
**720575940614418227**	Sm26	7

Table 3. Quantification of pDm8 (720575940638634960) inputs.

Flywire ID	cell type	pre sites (n)
**720575940636715998**	yR7	35
**720575940628506897**	yR7	15
**720575940660740481**	yR7	15
**720575940637440613**	yR7	8
**720575940636942447**	yR7	6
**720575940641088640**	yR7	3
**720575940621704942**	yR7	3
**720575940632586297**	pR7	15
**720575940623014689**	pR7	10
**720575940621705198**	pR7	4
**720575940615537314**	pR7	3
**720575940631563515**	pR7	3
**720575940618562594**	pR7	3
**720575940613737330**	pR7	13
**720575940614131119**	Dm5	18
**720575940630896379**	Dm5	9
**720575940630969303**	Dm	5
**720575940637975741**	Mi3	4
**720575940648106116**	Dm2	10
**720575940655698849**	Dm9	4
**720575940621192431**	Dm5	3
**720575940639473998**	Tm5a	3
**720575940623904136**	Mi3	8
**720575940627112344**	Mi10	7
**720575940615782978**	Mi10	3
**720575940622466036**	CB0566	21

**720575940628838474**	CB0171	8
**720575940626457893**	aME17b	7
**720575940621186219**	aMe17b	5
**720575940625414477**	aMe17b	4
**720575940615181479**	Sm19	20
**720575940633602017**	Sm13	4
**720575940633387181**	Sm19	6
**720575940641982709**	Sm20	12
**720575940615314834**	Sm20	10
**720575940628760561**	Sm19	9
**720575940605990590**	Sm19	5
**720575940624488445**	Sm20	6
**720575940613095282**	Sm20	10
**720575940634295010**	Sm21	3
**720575940622315647**	Sm20	4
**720575940625259704**	Sm20	4
**720575940627733777**	Sm20	7
**720575940633138771**	Sm20	7
**720575940617838225**	Sm26	8
**720575940614418227**	Sm26	9

Table 4. Quantification of yDm8 (720575940638424895) inputs.

Flywire ID	cell type	pre sites Me (n)	pre sites Lo (n)
**720575940636715998**	yR7	29	0
**720575940623904136**	Mi3	17	0
**720575940620683456**	Mi4	14	0
**720575940638424895**	yDm8	13	0
**720575940622664455**	Tm5a_like	9	0
**720575940614087087**	yDm8	7	0
**720575940614902495**	Tm5a	7	0
**720575940611231608**	Li19	0	6
**720575940621687649**	LT58	0	5
**720575940622762677**	Tm	5	0
**720575940623747818**	Tm5c	5	0
**720575940626457893**	aMe17b	4	0
**720575940627946056**	TmY5a	4	0
**720575940632492625**	L3	4	0
**720575940618141268**	Tm24	0	4
**720575940627314521**	Tm5c	4	0
**720575940629248771**	Tm	3	0
**720575940616587677**	TmY	3	0
**720575940646093166**	L3	3	0
**720575940632734924**	Tm5b	3	0
**720575940610787118**	TmY20	0	3

Table 5. Quantification of Tm5a (720575940639473998) inputs.

Flywire ID	cell type	pre sites Me (n)	pre sites Lo (n)

**720575940636942447**	yR7	24	0
**720575940614087087**	yDm8	26	0
**720575940619053873**	Li19	0	7
**720575940623040470**	Dm	6	0
**720575940626457893**	aMe17b	6	0
**720575940618913158**	Y3	4	0
**720575940620474315**	Tm	4	0
**720575940637975741**	Mi3	3	0
**720575940618200038**	LC20	0	3
**720575940636163832**	aMe17b	3	0
**720575940623904136**	Mi3	3	0
**720575940614686259**	Sm31	3	0
**720575940627406650**	Tm	3	0
**720575940633589677**	Tm	3	0
**720575940627946056**	TmY5a	3	0
**720575940616394694**	CB0566	3	0
**720575940616587677**	TmY	3	0

Table 6. Quantification of Tm5a (720575940614902495) inputs.

Flywire ID	cell type	pre sites Me (n)	pre sites Lo (n)
**720575940632586297**	pR7	20	0
**720575940624675079**	pR7	4	0
**720575940622269424**	pR8	2	0
**720575940625356011**	yR8	2	0
**720575940638634960**	pDm8	16	0
**720575940623113962**	Y3	10	1
**720575940623904136**	Mi3	11	0
**720575940618913158**	Y3	4	4
**720575940624951027**	pDm8	8	0
**720575940621453921**	Pm2	8	0
**720575940635247711**	Tm	7	0
**720575940639450165**	TmY5b	7	0
**720575940634254562**	Pm2	7	0
**720575940632473391**	TmY5b	6	0
**720575940619305374**	TmY5b	6	0
**720575940622757895**	Mi4	6	0
**720575940627314521**	Tm5c	6	0
**720575940619252603**	Tm5c	5	0
**720575940622508156**	Dm	5	0
**720575940660846721**	Tm5b	5	0
**720575940626924675**	TmY5a	5	0
**720575940632763975**	Sm26	5	0
**720575940633602017**	Sm13	5	0
**720575940636163832**	aMe17b	4	0
**720575940622560636**	Sm26	4	0
**720575940615657538**	Mi3	4	0
**720575940648130436**	Dm	4	0

**720575940626012208**	Mi10	4	0
**720575940626457893**	aMe17b	4	0
**720575940614620010**	Y3	2	1
**720575940616578594**	Y3	3	0
**720575940611157458**	TmY5b	3	0
**720575940614902495**	Tm5a	0	3
**720575940635684715**	Sm26	3	0
**720575940623672856**	Mi4	3	0
**720575940637755701**	Mi4	3	0
**720575940621771248**	Dm3	3	0
**720575940617838225**	Sm26	3	0
**720575940629822619**	Sm40	3	0
**720575940636604271**	Pm2	3	0
**720575940610378104**	Y3	2	1

Table 7. Quantification of Tm5b (720575940627282584) inputs.

Flywire ID	cell type	pre sites Me (n)	pre sites Lo (n)
**720575940632586297**	pR7	19	0
**720575940625479916**	yR8	6	0
**720575940634575329**	pR8	1	0
**720575940624951027**	pDm8	19	0
**720575940605362912**	Mi4	11	0
**720575940618913158**	Y3	9	1
**720575940619252603**	Tm5c	9	0
**720575940623904136**	Mi3	8	0
**720575940614620010**	Y3	5	1
**720575940627282584**	Tm5b	5	0
**720575940627946056**	TmY5a	5	0
**720575940615011490**	Mi3	5	0
**720575940614087087**	yDm8	5	0
**720575940626637002**	mALC2	0	5
**720575940616587677**	TmY	4	0
**720575940604973536**	T2	0	4
**720575940616578594**	Y3	0	4
**720575940626968146**	Li19	0	4
**720575940646658083**	TmY5b	4	0
**720575940630163959**	Mi1	4	0
**720575940632763975**	Sm26	3	0
**720575940629465499**	TmY5a	3	0
**720575940622485786**	Tm	0	3
**720575940629822619**	Sm40	3	0
**720575940619323806**	Mi4	3	0
**720575940635539374**	Mi9	3	0
**720575940627406650**	Tm	3	0
**720575940626331184**	Li12	0	3
**720575940626634789**	Li	0	3
**720575940623353284**	Sm13	3	0

**720575940618431798**	Y3	0	3

Table 8. Quantification of Tm5b (720575940660846721) inputs.

Flywire ID	cell type	pre sites Me (n)	pre sites Lo (n)
**720575940622269424**	pR8	6	0
**720575940615537314**	pR7	5	0
**720575940625356011**	yR8	4	0
**720575940634575329**	pR8	4	0
**720575940625479916**	yR8	4	0
**720575940618886228**	yR8	3	0
**720575940632586297**	pR7	2	0
**720575940621307483**	aMe17a	19	0
**720575940655090849**	L3	10	0
**720575940615325090**	aMe4	10	0
**720575940636455031**	L3	9	0
**720575940614575527**	L3	8	0
**720575940613737874**	MTe50	6	0
**720575940631627595**	cM08b	5	0
**720575940625062891**	Li13	0	5
**720575940611040046**	L3	5	0
**720575940609770691**	Mi4	4	0
**720575940627282584**	Tm5b	4	0
**720575940636501093**	Tm16	4	0
**720575940615143934**	CB0471	3	0
**720575940645690734**	TmY10	3	0
**720575940627110147**	Li19	0	3
**720575940613428246**	LoP	0	3
**720575940626012208**	Mi10	3	0
**720575940626175242**	Tm16	3	0
**720575940625278652**	aMe4	3	0
**720575940620200021**	Tm5b	0	3
**720575940614131119**	Dm	3	0
**720575940612877363**	Tm25	3	0
**720575940660846721**	Tm5b	3	0

Table 9. Quantification of Tm5c (720575940619252603) inputs.

Flywire ID	cell type	pre sites Me (n)	pre sites Lo (n)
**720575940625479916**	yR8	22	0
**720575940618886228**	yR8	21	0
**720575940636942447**	yR7	2	0
**720575940621307483**	aMe17a	26	0
**720575940632492625**	L3	20	0
**720575940636455031**	L3	14	0
**720575940625278652**	aMe4	13	0
**720575940655090849**	L3	11	0
**720575940613789411**	L3	10	0
**720575940618535425**	aMe4	10	0

**720575940614087087**	yDm8	9	0
**720575940620474571**	Mi	8	0
**720575940631424197**	Tm16	0	7
**720575940631009531**	TmY10	6	0
**720575940621453165**	CB0816	5	0
**720575940626084666**	TmY9	0	5
**720575940660477313**	Tm	5	0
**720575940626270000**	MTe50	4	0
**720575940614788519**	Tm	4	0
**720575940618438941**	aMe4	4	0
**720575940610028995**	CB0816	3	0
**720575940640940496**	L5	3	0
**720575940616121503**	Lt56	0	3
**720575940638571583**	Mi1	3	0
**720575940616335118**	unknown	3	0
**720575940632868177**	CB0816	3	0
**720575940626350794**	Tm	3	0

Table 10. Quantification of Tm5c (720575940627314521) inputs.

Flywire ID	cell type	pre sites Me (n)	pre sites Lo (n)
**720575940622269424**	pR8	32	0
**720575940632586297**	pR7	3	0
**720575940655090849**	L3	51	0
**720575940622757895**	Mi4	38	0
**720575940625657221**	Tm1	16	0
**720575940630796918**	L2	15	0
**720575940614620010**	Y3	6	3
**720575940632868177**	CB0816	9	0
**720575940623113962**	Y3	7	0
**720575940607136130**	Dm3	6	0
**720575940626637002**	mALC2	0	6
**720575940631893132**	unknown	5	0
**720575940646691363**	ML1	4	0
**720575940605835906**	unknown	4	0
**720575940621886068**	Sm31	4	0
**720575940632002616**	C3	4	0
**720575940628559366**	Tm20	4	0
**720575940630281516**	TmY5a	4	0
**720575940631354572**	T2 cell	3	0
**720575940618504395**	Mi1	3	0
**720575940640940496**	L5	3	0
**720575940655546017**	LC6	0	3
**720575940630394306**	Sm31	3	0
**720575940631191926**	Li12	0	3

Table 11. Quantification of Tm20 (720575940635252890) inputs.

Flywire ID	cell type	pre sites Me (n)	pre sites Lo (n)

**720575940618886228**	yR8	30	0
**720575940636715998**	yR7	1	0
**720575940632492625**	L3	36	0
**720575940620683456**	Mi4	34	0
**720575940623664422**	L2	14	0
**720575940632988205**	C3	12	0
**720575940612464114**	Tm1	9	0
**720575940614620010**	Y3	5	2
**720575940632167481**	Tm20	4	3
**720575940631191926**	Li12	0	6
**720575940622743209**	CB0816	5	0
**720575940630837551**	ML1	5	0
**720575940638571583**	Mi1	4	0
**720575940640228669**	Sm31	4	0
**720575940641642715**	Me4	4	0
**720575940635456430**	Sm22	4	0
**720575940625547452**	Sm22	4	0
**720575940635369663**	Sm22	4	0
**720575940623974122**	Sm22	3	0
**720575940630125562**	LT34	0	3
**720575940646691363**	ML1	3	0
**720575940618712093**	Li12	0	3
**720575940624556295**	T2a	3	0
**720575940621881328**	T2a	3	0
**720575940623394765**	T1	3	0
**720575940623747818**	Tm5c	0	3
**720575940636274551**	Tm20	3	0

Table 12. Quantification of Tm20 (720575940628559366) inputs.

Flywire ID	cell type	post sites (n)
**720575940627282584**	Tm5b	34
**720575940625193064**	Dm9	23
**720575940617259835**	Dm5	21
**720575940622692265**	Dm2	18
**720575940655698849**	Dm9	14
**720575940620346048**	TmY	14
**720575940630969303**	Dm4	11
**720575940660477313**	Tm5b_like	11
**720575940640326717**	Tm	10
**720575940634164124**	Dm5	9
**720575940612649625**	Dm2	9
**720575940615520827**	TmY	9
**720575940626924675**	TmY	8
**720575940619121472**	MC66	8
**720575940614418227**	Sm26	8
**720575940614131119**	Dm7	7
**720575940614788519**	Tm5c	7

**720575940626977768**	MC66	7
**720575940617838225**	Sm26	7
**720575940646634275**	Tm5a_like	6
**720575940635081567**	MTe02	6
**720575940641698523**	aMe12	6
**720575940628428821**	Dm4	5
**720575940622757895**	Mi4	5
**720575940630688256**	TmY	5
**720575940630652124**	MLt2	5
**N/A**	unknown	7
**N/A**	unknown	6

Table 13. Quantification of pDm8 (720575940638634960) outputs.

Flywire ID	cell type	post sites (n)
**720575940625193064**	Dm9	22
**720575940639473998**	Tm5a	22
**720575940621192431**	Dm5	21
**720575940635624696**	Dm4	21
**720575940640334208**	Tm5b	21
**720575940655698849**	Dm9	16
**720575940610288452**	TmY	16
**720575940627733777**	Sm20	14
**720575940622664455**	Tm5a_like	12
**720575940617707494**	Sm20	12
**720575940631460423**	MTe02	12
**720575940631776975**	Dm5	10
**720575940622762677**	Tm	10
**720575940609837717**	Sm20	10
**720575940633718623**	Tm5a	8
**720575940623904136**	Mi3	8
**720575940623406605**	CB0156	8
**720575940631345751**	MC61	8
**720575940613095282**	Sm20	8
**720575940586278879**	unknown	8
**720575940647395636**	MLt6	7
**720575940635081567**	MTe02	7
**720575940612387251**	MLt6	7
**720575940632547129**	Sm19	7
**720575940628838474**	CB0171	6
**720575940625292839**	Sm19	6
**720575940631458575**	Sm19	6
**720575940616130975**	TmY	6
**720575940644657608**	MeMe_e09	6
**720575940629626455**	Tm	6
**720575940621025262**	aMe9	6
**720575940660477313**	Tm5b_like	6
**720575940620398040**	Tm5b	6

**720575940612047858**	Tm5c	5
**720575940634164124**	Dm5	5
**720575940635648191**	Sm41	5
**720575940623071256**	Mi15	5

Table 14. Quantification of yDm8 (720575940638424895) outputs.

Flywire ID	cell type	post sites Me (n)	post sites Lo (n)
**720575940626637002**	mALC2	0	25
**720575940617134779**	unknown	0	18
**720575940616109930**	LC10c	0	16
**720575940615579498**	Li1	0	12
**720575940615249730**	LC10c	0	11
**720575940618141268**	Tm24	0	8
**720575940626577490**	LC26	0	7
**720575940623406605**	CB0156	7	0
**720575940628991682**	LC24	0	5
**720575940609078997**	LT87	0	5
**720575940608617564**	LPLC2	0	5
**720575940629048108**	Tm24	0	5
**720575940627946056**	Tm5Y	0	5
**720575940640334208**	Tm	5	0
**720575940615045630**	Li1	0	5
**720575940640228669**	Sm31	5	0

Table 15. Quantification of Tm5a (720575940639473998) outputs.

Flywire ID	cell type	post sites Me (n)	post sites Lo (n)
**720575940626637002**	mALC2	0	16
**720575940610786862**	unknown	0	14
**720575940618200038**	LC20	0	13
**720575940624919079**	LC37	0	10
**720575940623406605**	CB0156	9	0
**720575940623757543**	LC26	0	9
**720575940615522666**	unknown	0	9
**720575940628296527**	Tm5b	8	0
**720575940639473998**	Tm5a	7	0
**720575940633968538**	LC10c	0	7
**720575940631148428**	Li2	0	7
**720575940620478032**	unknown	0	7
**720575940626350794**	Tm	6	0
**720575940630642495**	Sm19	6	0
**720575940588309383**	unknown	0	6
**720575940630589655**	TmY	0	5
**720575940621886068**	Sm31	5	0
**720575940618141268**	Tm24	0	5
**720575940596370762**	unknown	0	5

Table 16. Quantification of Tm5a (720575940614902495) outputs.

Flywire ID	cell type	post sites Me (n)	post sites Lo (n)
**720575940625550823**	Sm40	22	0
**720575940638458173**	LC6	0	20
**720575940655546017**	LC6	0	19
**720575940620200021**	Tm5b	8	5
**720575940623904136**	Mi3	9	0
**720575940628838474**	CB0171	8	0
**720575940614344429**	Sm40	7	0
**720575940631148428**	Li2	0	6
**720575940626264201**	unknown	6	0
**720575940612227441**	LC6	0	6
**720575940643962903**	Tm5b	4	1
**720575940622540894**	Sm21	5	0
**720575940626012208**	Mi10	5	0
**720575940660846721**	Tm5b	5	0
**720575940634254562**	Pm4	5	0
**720575940593785522**	unknown	5	0
**720575940591584450**	unknown	0	5
**720575940639893301**	Sm40	5	0

Table 17. Quantification of Tm5b (720575940627282584) outputs.

Flywire ID	cell type	post sites Me (n)	post sites Lo (n)
**720575940638458173**	LC6	0	17
**720575940628838474**	CB0171	11	0
**720575940620200021**	Tm5b	12	0
**720575940655546017**	LC6	0	9
**720575940617678553**	Li2	0	9
**720575940620283608**	Li3	0	9
**720575940614344429**	Sm40	8	0
**720575940609770691**	Mi4	8	0
**720575940621301857**	Li3	0	7
**720575940626012208**	Mi10	7	0
**720575940619252718**	LC17	0	7
**720575940639893301**	Sm40	7	0
**720575940502731931**	unknown	0	6
**720575940608610652**	LC17	0	6
**720575940615790146**	unknown	0	5
**720575940627282584**	Tm5b	5	0
**720575940636206558**	LC13	0	5
**720575940640370048**	LC10b	0	5
**720575940631148428**	Li2	0	5
**720575940628495622**	Li3	0	5
**720575940628662377**	Mi10	5	0

Table 18. Quantification of Tm5b (720575940660846721) outputs.

Flywire ID	cell type	post sites Me (n)	post sites Lo (n)
**720575940623777577**	Tm	23	0

**720575940639893301**	Sm40	22	0
**720575940616951586**	Mi15	16	0
**720575940624832008**	Dm	16	0
**720575940633457107**	Mi15	13	0
**720575940634254562**	Pm2	12	0
**720575940621453921**	Pm2	11	0
**720575940644124832**	Mi	10	0
**720575940620200021**	Tm5b	10	0
**720575940612927385**	Dm12	10	0
**720575940660846721**	Tm5b	9	0
**720575940622692265**	Dm	8	0
**720575940640427904**	unknown	8	0
**720575940612877363**	Tm	6	1
**720575940626350794**	Tm	2	5
**720575940599503195**	unknown	7	0
**720575940621386849**	Tm	6	0
**720575940621804353**	Dm20	6	0
**720575940633891042**	unknown	6	0
**720575940627551631**	Tm	6	0
**720575940557718029**	unknown	6	0
**720575940630649911**	Y4	1	5
**720575940644125600**	Mi2	6	0
**720575940631448121**	Tm	5	0
**720575940628898890**	Mi15	5	0
**720575940631148428**	Li2	0	5
**720575940623013325**	T2a	5	0
**720575940627282584**	Tm5b	5	0

Table 19. Quantification of Tm5c (720575940619252603) outputs.

Flywire ID	cell type	post sites Me (n)	post sites Lo (n)
**720575940623877560**	Tm	28	5
**720575940626350794**	Tm	20	1
**720575940639893301**	Sm40	17	0
**720575940614788519**	Tm	15	0
**720575940621339886**	Mi15	14	0
**720575940625234020**	Mi15	14	0
**720575940634254562**	Pm2	12	0
**720575940624832008**	Dm2	8	0
**720575940630559299**	Dm2	8	0
**720575940622725341**	Dm2	8	0
**720575940660846721**	Tm5b	2	5
**720575940628898890**	Mi15	7	0
**720575940641897101**	unknown	6	0
**720575940630054647**	Dm	6	0
**720575940627807492**	Mi4	6	0
**720575940627282584**	Tm5b	6	0
**720575940633891042**	unknown	5	0

**720575940626997770**	unknown	5	0
**720575940622561356**	Mi15	5	0
**720575940626766224**	unknown	5	0
**720575940632877004**	aMe5	5	0
**720575940639795133**	mALC1	0	5

Table 20. Quantification of Tm5c (720575940627314521) outputs.

Flywire ID	cell type	post sites Me (n)	post sites Lo (n)
**720575940621535809**	LC16	0	17
**720575940622664455**	Tm5a_like	16	0
**720575940617914693**	LC16	0	14
**720575940626924675**	Tm5a_like	14	0
**720575940611711461**	LC10a	0	12
**720575940615045630**	Li1	0	10
**720575940610786862**	unknown	0	9
**720575940628991682**	LC24	0	8
**720575940605835906**	unknown	8	0
**720575940615249730**	LC10c	0	7
**720575940613083609**	unknown	7	0
**720575940631007183**	Tm	7	0
**720575940627946056**	Tm5Ya	0	7
**720575940627978152**	LC10c	0	7
**720575940632002616**	C3	6	0
**720575940649953913**	LC22	0	6
**720575940638733621**	unknown	6	0
**720575940612530905**	unknown	6	0
**720575940626605657**	unknown	6	0
**720575940635658420**	Tm	6	0
**720575940621886068**	Sm31	6	0
**720575940629110019**	Li3	0	6
**720575940628898665**	Tm	5	0
**720575940630281516**	Tm5Y	5	0
**720575940638655972**	MTe08	5	0
**720575940637952846**	LC16	0	5
**720575940637308528**	Mi9	5	0
**720575940623051725**	Tm	5	0
**720575940613503510**	Sm22	5	0
**720575940631893132**	unknown	5	0
**720575940626163837**	MC62	5	0

Table 21. Quantification of Tm20 (720575940635252890) outputs.

Flywire ID	cell type	post sites Me (n)	post sites Lo (n)
**720575940632039213**	Tm5Ya	15	0
**720575940610786862**	unknown	0	11
**720575940628898665**	Tm	10	0
**720575940626163837**	MC62	9	0
**720575940611711461**	LC10a	0	9

**720575940615045630**	Li1	0	9
**720575940611896434**	Ml9	8	0
**720575940639841870**	Tm	8	0
**720575940619726507**	LC24	0	8
**720575940628296527**	Tm	7	0
**720575940609666499**	Tm	7	0
**720575940621535809**	LC16	0	7
**720575940625701769**	LC16	0	6
**720575940632145720**	Tm13Y	6	0
**720575940622664455**	Tm5a_like	6	0
**720575940623393048**	TmY	6	0
**720575940637952846**	LC16	0	5
**720575940626248325**	Sm22	5	0
**720575940606410439**	unknown	0	5
**720575940602874055**	unknown	5	0
**720575940628494535**	LC28b	0	5
**720575940626378128**	MTe03	5	0
**720575940615249730**	LC10c	0	5
**720575940627946056**	Tm5Ya	5	0

Table 22. Quantification of Tm20 (720575940628559366) outputs.

Figure	Genotype	Genotype ID
**Figure 1**		
**Figure 1g**	w+; Rh4-Gal4/+; 20xUAS-GCaMP6f/+;	518
	w+; Rh3-Gal4/+; 20xUAS-GCaMP6f/+;	517
	w+; 20xUAS-GCaMP6f/+; Rh6-Gal4/+;	529
	w+; Rh5-Gal4/+; 20xUAS-GCaMP6f/+;	519
	w+; OrtC1a-Gal4.DBD, 20XUAS-GCaMP6f/+; ET24gdVP16.AD/+;	1191
	w+; 27E03-p65.AD/+; 94H07-Gal4.DBD, 20xUAS-GCaMP6f/+;	1428
	w+; OrtC1a-Gal4.DBD,OK371-Vp16.AD/+; 20xUAS-GCaMP6f/+;	952
	w+; R41E03-p65.AD/20xUAS-GCaMP6f; R81G11-Gal4.DBD/+;	1929
**Figure 2**		
**Figure 2a, i-k**	w+; Rh4-Gal4/+; 20xUAS-GCaMP6f/+;	518
**Figure 2b, i-k**	w+; Rh3-Gal4/+; 20xUAS-GCaMP6f/+;	517
**Figure 2c, i-k**	w+; 20xUAS-GCaMP6f/+; Rh6-Gal4/+;	529
**Figure 2d, i-k**	w+; Rh5-Gal4/+; 20xUAS-GCaMP6f/+;	519
**Figure 2e, i-k**	w+; 27E03-p65.AD/+; 94H07-Gal4.DBD, 20xUAS-GCaMP6f/+;	1428
**Figure 2f, i-k**	w+; OrtC1a-Gal4.DBD, 20XUAS-GCaMP6f/+; ET24gdVP16.AD/+;	1191
**Figure 2g, i-k**	w+; OrtC1a-Gal4.DBD,OK371-Vp16.AD/+; 20xUAS-GCaMP6f/+;	952
**Figure 2h, i-k**	w+; R41E03-p65.AD/20xUAS-GCaMP6f; R81G11-Gal4.DBD/+;	1929
**Figure 4**		
**Figure 4l**	w+; 27E03-p65.AD/UAS-TeTxLC.tnt; 94H07-Gal4.DBD, 20xUAS-GCaMP6f/+;	2368
**Figure 4m**	w+; OrtC1a-Gal4.DBD, 20XUAS-GCaMP6f/UAS-TeTxLC.tnt; ET24gdVP16.AD/+;	2293
**Figure 4n**	w+; OrtC1a-Gal4.DBD,OK371-Vp16.AD/UAS-TeTxLC.tnt; 20xUAS-GCaMP6f/+;	2547
**Figure 4o**	w+; OrtC1a-Gal4.DBD, touVP16.AD/UAS-TeTxLC.tnt; 20xUAS-GCaMP6f/+;	2511

**Figure S1**		
**Figure S1a, j, s**	w+; Rh4-Gal4/+; 20xUAS-GCaMP6f/+;	518
**Figure S1b, k, t**	w+; Rh3-Gal4/+; 20xUAS-GCaMP6f/+;	517
**Figure S1c, l, u**	w+; 20xUAS-GCaMP6f/+; Rh6-Gal4/+;	529
**Figure S1d, m, v**	w+; Rh5-Gal4/+; 20xUAS-GCaMP6f/+;	519
**Figure S1e, n, w**	w+; 27E03-p65.AD/+; 94H07-Gal4.DBD, 20xUAS-GCaMP6f/+;	1428
**Figure S1f, o, x**	w+; OrtC1a-Gal4.DBD, 20XUAS-GCaMP6f/+; ET24gdVP16.AD/+;	1191
**Figure S1g, p, y**	w+; OrtC1a-Gal4.DBD,OK371-Vp16.AD/+; 20xUAS-GCaMP6f/+;	952
**Figure S1h, q, z**	w+; R41E03-p65.AD/20xUAS-GCaMP6f; R81G11-Gal4.DBD/+;	1929
**Figure S2**		
**Figure S2a**	w+; Rh3-Gal4/+; 20xUAS-GCaMP6f/+;	517
**Figure S2b**	w+; Rh4-Gal4/+; 20xUAS-GCaMP6f/+;	518
**Figure S3**		
**Figure S3d, h**	w+; 27E03-p65.AD/+; 94H07-Gal4.DBD, 20xUAS-GCaMP6f/+;	1191
**Figure S3e, i**	w+; OrtC1a-Gal4.DBD, 20XUAS-GCaMP6f/+; ET24gdVP16.AD/+;	1428
**Figure S3f, j**	w+; OrtC1a-Gal4.DBD,OK371-Vp16.AD/+; 20xUAS-GCaMP6f/+;	952
**Figure S3g, k**	w+; R41E03-p65.AD/20xUAS-GCaMP6f; R81G11-Gal4.DBD/+;	1929
**Figure S6**		
**Figure S6a, c, e, g-i, k, l**	w+; 20XUAS-GCamp6f/+; OrtC2b-Gal4,DIPγ-Gal80/+;	830
**Figure S6b, d, f, g-i, j, m**	w+; OrtC1-3-Vp16.AD/20xUAS-GCaMP6f; DIPγ-Gal4.DBD/+;	777
**Figure S7**		
**Figure S7n**	w+; 27E03-p65.AD/UAS-TeTxLC.tnt; 94H07-Gal4.DBD, 20xUAS-GCaMP6f/+;	2368
**Figure S7o**	w+; OrtC1a-Gal4.DBD, 20XUAS-GCaMP6f/UAS-TeTxLC.tnt; ET24gdVP16.AD/+;	2293
**Figure S7p**	w+; OrtC1a-Gal4.DBD,OK371-Vp16.AD/UAS-TeTxLC.tnt; 20xUAS-GCaMP6f/+;	2547
**Figure S7q**	w+; OrtC1a-Gal4.DBD, VP16.AD.tou-9A30/UAS-TeTxLC.tnt; 20xUAS-GCaMP6f/+;	2511
**Figure S9**		
**Figure S9a, b**	R57C10-FLPG5.PEST/y-w-; 27E03-p65.AD/+; 94H07-Gal4.DBD, 20xUAS-GCaMP6f/ 10xUAS(FRT.stop)myr::smGdP-HA, 10xUAS(FRT.stop)myr::smGdP-V5-THS-10xUAS(FRT.stop)myr::smGdP-FLAG	N/A

Table 23. Genotypes used in each figure
